# Melting Temperature Mapping Method: A Novel Method for Rapid Identification of Unknown Pathogenic Microorganisms within Three Hours of Sample Collection

**DOI:** 10.1038/srep12543

**Published:** 2015-07-28

**Authors:** Hideki Niimi, Tomohiro Ueno, Shirou Hayashi, Akihito Abe, Takahiro Tsurue, Masashi Mori, Homare Tabata, Hiroshi Minami, Michihiko Goto, Makoto Akiyama, Yoshihiro Yamamoto, Shigeru Saito, Isao Kitajima

**Affiliations:** 1 Clinical Laboratory Center, Toyama University Hospital, Toyama 930-0194, Japan; 2 Kitami Information Technology Co., Ltd., Hokkaido 090-0813, Japan; 3 Research Institute for Bioresources and Biotechnology, Ishikawa Prefectural University, Ishikawa 921-8836, Japan; 4 Life Science Center, Hokkaido Mitsui Chemicals, Inc., Hokkaido 073-0138, Japan; 5 Department of Internal Medicine, University of Iowa Carver College of Medicine, IA 52242, USA, and Iowa City Veterans Affairs Medical Center, IA 52246, USA; 6 Nagaresugi Geriatric Hospital, Toyama 939-8032, Japan; 7 Department of Clinical Infectious Diseases, Toyama University Hospital, Toyama 930-0194, Japan; 8 Department of Obstetrics & Gynecology, Toyama University Hospital, Toyama 930-0194, Japan

## Abstract

Acquiring the earliest possible identification of pathogenic microorganisms is critical for selecting the appropriate antimicrobial therapy in infected patients. We herein report the novel “melting temperature (Tm) mapping method” for rapidly identifying the dominant bacteria in a clinical sample from sterile sites. Employing only seven primer sets, more than 100 bacterial species can be identified. In particular, using the Difference Value, it is possible to identify samples suitable for Tm mapping identification. Moreover, this method can be used to rapidly diagnose the absence of bacteria in clinical samples. We tested the Tm mapping method using 200 whole blood samples obtained from patients with suspected sepsis, 85% (171/200) of which matched the culture results based on the detection level. A total of 130 samples were negative according to the Tm mapping method, 98% (128/130) of which were also negative based on the culture method. Meanwhile, 70 samples were positive according to the Tm mapping method, and of the 59 suitable for identification, 100% (59/59) exhibited a “match” or “broad match” with the culture or sequencing results. These findings were obtained within three hours of whole blood collection. The Tm mapping method is therefore useful for identifying infectious diseases requiring prompt treatment.

Acquiring the earliest possible identification of pathogenic microorganisms is critical for selecting the appropriate antimicrobial therapy and obtaining a favorable outcome in infected patients^1^,^2^,^3^. Severe systemic infections, such as sepsis, are a primary cause of morbidity and mortality in hospitalized patients, the definitive diagnosis of which requires proper identification of the causative microorganism^4^. However, as current pathogen-identification methods using microbial cultures require several days, empirically selected antimicrobial agents are often administered until the pathogenic microbes are identified^5^,^6^. As a result, the use of inappropriate antimicrobial agents in the initial treatment period often results in life-threatening conditions in patients with severe infections^7^. In addition, the overuse of broad-spectrum antimicrobial agents has led to the emergence of drug-resistant bacteria. Hence, there are significant risks associated with the initial treatment of infectious diseases and there is thus a critical need to develop new methods capable of rapidly identifying pathogenic microorganisms. Such methods would make possible the more informed use of antimicrobial agents at an earlier stage and consequently reduce the inappropriate overuse of broad-spectrum antimicrobial agents and slow the emergence of more drug-resistant bacteria^8^. In this way, the rapid identification of pathogenic microorganisms is a powerful tool for optimizing antibiotic stewardship. For clinicians, the clock starts long before the growth of bacterial colonies, beginning with patient sample collection. As long as microbial cultures are used, it is difficult to establish a rapid system because the speed of detection depends on the growth rate of the bacterial species. In this regard, even mass spectrometry-based identification, which at present also require microbial culture, is no exception^9^,^10^.

In order to identify pathogens more rapidly, genetic testing methods have been developed for non-culture diagnosis. Identification of the pathogenic species can be achieved using a range of molecular genetic techniques, including multiplexing^11^, hybridization probes^12^, microarrays^13^ and gene sequencing^14^. However, the number of pathogens capable of being identified using multiplexing and hybridization probes is limited, as the usable number of species-specific primers or probes is also limited technically. In addition, microarrays cannot quickly adapt to the emergence of mutant strains of bacteria and both microarrays and gene sequencing analyses tend to be costly. These techniques therefore have limited applications for identifying infectious diseases in clinical practice.

In an attempt to address these problems, we herein report the development of a novel rapid, easy and cost-effective method for identifying a broad range of pathogenic bacteria using a real-time PCR-based system. Employing only seven primer sets, more than 100 bacterial species can be rapidly identified without the need for microbial culture, multiplexing, hybridization probes or gene sequencing, and the number of identifiable bacterial species is easily expandable.

## Results

### Workflow of the novel rapid identification method

**Figure 1:**
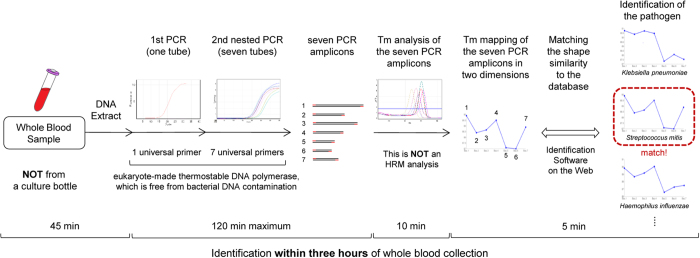
Workflow of the novel rapid method for identifying unknown pathogenic bacteria within three hours of whole blood collection.

The workflow of the novel rapid identification method for unknown pathogenic bacteria developed in our laboratory is shown in Fig. 1. Within three hours of whole blood collection, this method identifies the dominant bacteria, which is usually pathogenic, in a clinical sample. This method consists of four major steps. First, bacterial DNA is extracted directly from a clinical sample (2 mL of a whole blood sample, etc.) as a template for PCR. Step two involves a nested PCR using seven bacterial universal primer sets (one primer set per tube in the second PCR); these primers can detect almost all species of bacteria. In order to achieve accuracy in this PCR step, we developed a eukaryote-made thermostable DNA polymerase that is free from bacterial DNA contamination^15^. The eukaryote-made thermostable DNA polymerase is a recombinant polymerase manufactured using eukaryotic (yeast) host cells. Employing this DNA polymerase, sensitive and reliable detection of bacteria without false-positive results is feasible, thereby making it possible for PCR to identify bacterial isolates directly from patient samples. The nested PCR procedure is performed and seven (or less) PCR amplicons are obtained. If none of the seven amplifications are observed by the 30th cycle in the second PCR, then we defined the sample as having no bacteria present. In step three, the seven melting temperature (Tm) values are acquired by analyzing the amplicons. Step four involved mapping the seven Tm values on two dimensions (see Fig. 1). The plot creates a unique species-specific shape: the Tm mapping shape. This is NOT a high-resolution melting-curve (HRM) analysis, and only the Tm values were recorded. By comparing the Tm mapping shape to the shapes in the database, the bacterial isolates can be rapidly identified. We named this novel method the “Tm mapping method.” In order to make this method accessible globally, we developed an identification software program available on the World Wide Web.

### Concept of the Tm mapping method

**Figure 2:**
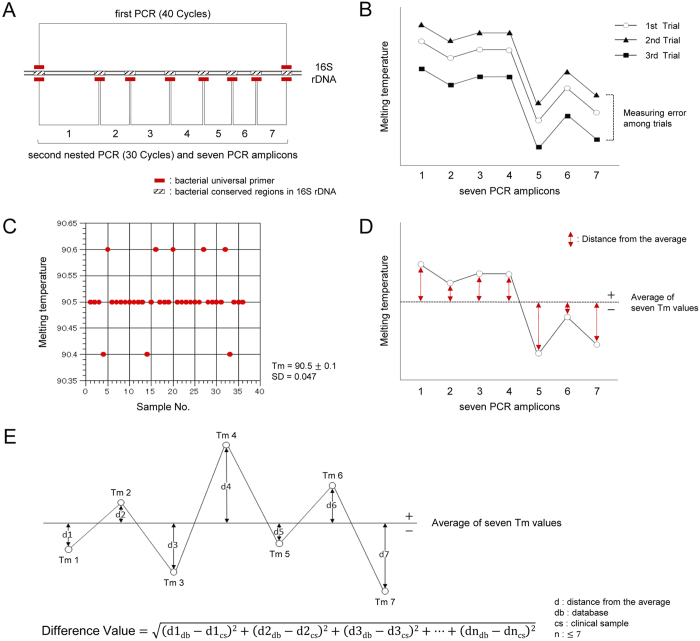
Concept of the Tm mapping method. (**A**) The strategy for the primer designs is shown. Nested PCR is performed using seven bacterial universal primer sets, and then the seven Tm values are obtained. (**B**) Mapping the seven Tm values on two dimensions leads to the identification of the unique bacterial species-specific shape. The average of all seven Tm values includes the measurement error among trials; however, the Tm mapping shape is not affected by this type of error. (**C**) Using an analytical instrument with a high degree of thermal accuracy among PCR tubes and Tm value analysis with EvaGreen dye in 36 samples of the same bacterial DNA in the same trial, the tube-to-tube variation is within ±0.1 °C. (**D**) In order to analyze the Tm mapping “shape”, we developed a method to measure the distance of each individual Tm value from the average value. Tm values above the average receive a “+” designation, while those below the average receive a “−” designation. The Tm mapping shape is identified by comparing the seven distances obtained from the unknown bacteria to those in the database. (**E**) In order to identify a bacterial isolate, the identification software program calculates the Difference Values using the indicated formula. The closer the Difference Value is to zero, the more similar the Tm mapping shape is to the shape of a given species of pathogenic bacteria in the database.

The strategy for the primer designs is shown in Fig. 2A. In order to detect a wide range of bacteria, we designed seven bacterial universal primer sets targeting bacterial conserved regions in 16S ribosomal RNA gene (rDNA)^16^. We then devised a nested PCR assay to detect and identify bacterial isolates with high sensitivity and specificity.

Almost all measured Tm values contain measurement errors caused by the instrument. There are two types of measurement errors: measurement error among trials and measurement error among PCR tubes within the same trial (tube-to-tube variation). Measurement error among trials affects each Tm value equally, such that the overall Tm mapping shape is not affected by this kind of error (Fig. 2B). Tube-to-tube variation, however, is a serious issue, because it affects the Tm mapping shape. In order to minimize this variation, we decided to use an analytical instrument with a high degree of thermal accuracy among PCR tubes and performed the Tm value analysis with EvaGreen dye (Biotium, Inc.). Employing EvaGreen dye, it is possible to obtain stable Tm values, thereby minimizing the deviation error associated with tube-to-tube variation. Using an optimal instrument (Rotor-Gene Q by QIAGEN or LightCycler® Nano by Roche Applied Science) and the Tm value analysis with EvaGreen dye in 36 samples of the same bacterial DNA in the same trial, the tube-to-tube variation was within ±0.1 °C (standard deviation = 0.047) (Fig. 2C), which has little effect on the Tm mapping shape of the bacterial isolates, allowing for accurate identification.

In order to analyze the Tm mapping “shape”, we developed a method to measure the distance of each individual Tm value from the average value (Fig. 2D). Tm values above the average receive a “+” designation, while those below the average receive a “−” designation. These distances are not affected by the measurement error among trials. The Tm mapping shape is identified by comparing the seven distances obtained from unknown bacteria to those in the database.

Using the identification software program available on the World Wide Web, any user can identify bacterial isolates rapidly and easily without additional training (Fig. S1). In order to identify a bacterial isolate, the identification software program calculates the Difference Values using the formula shown in Fig. 2E. The Difference Value reflects the difference between the Tm mapping shape and that observed in the database. The closer the Difference Value is to zero, the more similar the Tm mapping shape is to the shape of the pathogenic bacteria contained in the database. From a mathematic point of view, the Difference Value is the distance between two points in seven dimensions. However, mapping the shape onto two dimensions provides much more information about each amplicon at a glance. Therefore, we decided to use the concept of the Tm mapping method. If the tube-to-tube variation is within ±0.1 °C, then the measurement error in the Difference Values is within 0.26 calculated by the Difference Value formula: 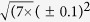), which should be taken into consideration when assessing the Tm mapping results.

### Construction of the Tm mapping database

Using the mean of triplicate Tm value measurements, we constructed a preliminary database with the Tm mapping shapes of 107 species, all of which are bacterial species detected in our hospital within the past five years (Fig. S2). The bacteria were obtained from clinical samples, then sequenced and identified to the species level. We also registered mutant strains (1 to 4 mutant strains per species) of the same bacterial species if the Tm mapping shape was a bit different compared with the shape in the database. The database is scalable and can be easily modified and updated. The individual Tm mapping shapes in the database show unique shapes reflecting the base sequence of each bacterial strain. Some Tm mapping shapes are missing data points; this is due to the fact that such Tm values cannot be obtained because the primers do not bind to their target regions. The primers binding to the bacterial target regions registered in the database are shown in Table S1. When the cycle threshold (Ct) is delayed for 10 or more cycles compared to the other primers in the amplification plot, we defined such primers as being unbound based on the low sequence homology between the universal primers and the bacterial target regions (Table S2). In order to identify the bacterial isolate, the identification software program narrows the scope of its search to bacteria in the database with the same pattern of primer binding in addition to calculating the Difference Values. That is, the pattern of the primer binding is also a characteristic of the bacteria.

### Assessment of the accuracy of the Tm mapping method

In order to assess the accuracy of the Tm mapping method, we first performed blind tests using the same 107 species of bacterial DNA registered in the database. That is, concealing the name of the bacteria, we tried to identify the bacterial DNA. In all 107 trials, the Tm mapping results matched the pre-sequenced bacterial DNA. The mean Difference Value was 0.178, with a range of 0.06 to 0.28 (standard deviation = 0.05).

Next, we set preliminary interpretative criteria to assess the suitability of the Tm mapping identification. The interpretative criteria for identification based on the Difference Value are shown in Table 1. Considering the range of Difference Values in the blind test, all test isolates with a Difference Value less than or equal to 0.28 have the same possibility of being the bacterial isolate (given that the theoretical error is 0.26, as described above). For this reason, if the Tm mapping method identified two or more species of bacteria with a Difference Value less than or equal to 0.28, the results cannot be narrowed down to one species, and we concluded that one of these bacterial species is the isolate. In this case, if one of these species matched the culture/sequencing results in our experiments, we defined the result as a “broad match”. If the Tm mapping method shows identification results with Difference Values greater than 0.28 and less than or equal to 0.5, the identification result with the lowest Difference Value is highly likely to be the bacterial isolate. In this case, the bacterial isolate would be either a mutant strain or a polymicrobial infection with one dominant bacterial species. If the Tm mapping method identifies a bacterial species with a Difference Value greater than 0.5, the result is not suitable for identification due to the low specificity of the Tm mapping identification (Table S3). In these cases, we do not try to identify the bacterial isolate and instead conclude only that bacteria are present.

We then validated the measurement error among three different instruments and trials using the same *Escherichia coli* (ATCC25922) DNA template (Table S4). Because the Tm mapping identification requires a tube-to-tube variation of no more than 0.1 °C within the same trial, we selected optimal instruments, such as the RotorGeneQ or LightCycler® Nano. The mean Difference Values of the validation procedures were as follows: first instrument (RotorGeneQ) = 0.191, second instrument (RotorGeneQ) = 0.164 and third instrument (LightCycler® Nano) = 0.199. All Difference Values were less than or equal to 0.28. We also assessed the range of Difference Values among 15 different trials starting from DNA extraction of the same *Escherichia coli* (ATCC25922) (Table S5). The mean Difference Value was 0.179, with a range of 0.13 to 0.26, which indicated that the reproducibility of the Tm mapping identification is stable from trial to trial.

Using the current protocols, the limit of identification is as follows (Table S6): *Escherichia coli* = 1.25 CFU/PCR tube (62.5 CFU/mL), *Staphylococcus aureus* = 1.25 CFU/PCR tube (62.5 CFU/mL), and *Klebsiella pneumoniae* = 0.63 CFU/PCR tube (31.3 CFU/mL). The limit of identification was determined to be the final log_2_ dilution of the template in which the Tm mapping result was correct, with the correct number of PCR amplicons (Table S1) and a Difference Value less than or equal to 0.5. Around the limit of identification, adequate amplification (melting peak curve) could not be obtained, therefore some of the Tm values could not be measured accurately (the Difference Values were greater than 0.28, but still within identification range).

We subsequently evaluated the accuracy of the Tm mapping method versus the culture/sequencing method (Table 2) using 140 bacterial colonies (51 bacterial species, shown in Table S7) obtained from various types of samples collected from different patients in chronological order of reception. Excluding 10 colonies not suitable for Tm mapping identification because the Difference Value was greater than 0.5, 98% (128/130) of the Tm mapping results were found to be either a “match” or “broad match” in comparison to the sequencing results. Concerning the accuracy of the culture results of these 130 samples, 93% (121/130) matched the sequencing results. All of the broad matches with sequencing results belonged to the genus *Staphylococcus* (Table S8). In this trial, all samples with Difference Values of greater than 0.5 occurred because the colonies contained more than one type of bacteria, although our intent was to test monomicrobial colonies. Some colonies had mutations in the 16S rDNA, so their Difference Values were higher than usual (0.28 < Difference Value ≤0.5). Whenever mutant strains are found, we make it a rule to register these strains in the database.

Finally, using 200 whole blood samples randomly collected from patients with suspected sepsis, we assessed the accuracy of the Tm mapping method compared that of the conventional culture method (Table 3). Of a total of 200 patient samples, 85% (171/200: A + D/Total) matched the culture results at the detection level (+ and −). Of the 130 samples negative according to the Tm mapping method, 98% (128/130) were also negative based on the culture method. Here, the culture method identified two types of bacteria that the Tm mapping method failed to detect. Using real whole blood samples the culture method identified *Staphylococcus epidermidis* and *Bacillus cereus*. We concentrated the DNA solution and tried to detect bacteria again however no bacteria were identified in either sample.

Seventy samples were positive according to the Tm mapping method, of which 43 were also positive using the culture method. In these 43 samples, 41 identifications matched the culture results, and two samples were not suitable for identification due to polymicrobial infections (Difference Value >0.5). Of the 27 samples found to be positive based on the Tm mapping method and negative based on the culture method, 18 could be identified, whereas the other nine could not.

The individual Tm mapping results compared with the culture or sequencing results are shown in Table 4. If the Tm mapping result did not match the culture result, we checked it again using the sequencing method. Of a total of 59 Tm mapping results suitable for Tm mapping identification (Difference Value ≤0.5), 100% (59/59) of the Tm mapping results were a “match” or “broad match” with the culture or sequencing results. For example, in patient No.4, the culture results showed both *Streptococcus agalactiae* and *Escherichia coli*, while Tm mapping and sequencing results showed only *Streptococcus agalactiae.* This is because the Tm mapping method is able to identify only the dominant bacteria in a clinical sample. In addition, in patient No. 34, the Tm mapping result was *Staphylococcus haemolyticus* or *Staphylococcus lugdunensis* because both Different Values were less than 0.28, whereas the culture and sequencing results were *Staphylococcus haemolyticus*.

## Discussion

The Tm mapping method can be used to identify bacterial isolates by mapping the unique shape of seven Tm values on two dimensions. This unique shape reflects the different DNA base sequences present among bacterial species. The Tm is defined as the temperature at which 50% of the double-stranded DNA dissociates into single strands. Based on the nearest neighbor thermodynamic theory, both the guanine and cytosine (GC) content of the DNA molecule and the specific nucleotide sequence has an effect on the Tm value^17^,^18^. For this reason, the Tm values provide adequate diversity for the Tm mapping method to identify many species of bacteria at the species level. The Tm mapping method does not require either bacterial cultures or sequencing analyses, thus permitting the rapid, easy and less expensive identification of bacterial isolates.

The Tm mapping method identifies the dominant bacteria in a clinical sample. Because no cultures are used, the number of bacteria in a clinical sample is accurately reflected, such that a small amount of contaminating bacteria can be distinguished using real-time PCR-based quantification. Depending on how quickly the dead bacteria are scavenged from the bloodstream^19^, rapid identification and real-time PCR-based quantification of the dominant bacteria may also make it possible to monitor the effects of treatment over time. In Table 4, in patients No. 4, No. 29 and No. 45, the Tm mapping method could not identify the bacterial isolates other than the dominant bacteria in polymicrobial infection, which is a weak point of this method. However, monitoring the effect of treatment, the Tm mapping method may identify the subdominant bacterial isolates because of microbial substitution induced by antibiotics. If the sample contains similar amounts of two or more species of bacteria, the Tm mapping method cannot identify the bacterial isolate because the different Tm values overlap (Fig. S3) and we subsequently conclude only that bacteria are present. In this regard, using the Difference Value, it is possible to quickly identify samples suitable for Tm mapping identification. If the Difference Value is greater than 0.5, then the result is not suitable for identification and there are three interpretations: a) there is a polymicrobial infection with no dominant species or b) the bacteria is not registered in the database or c) the bacterial concentration in the sample is under the limit of identification (Table 1). Polymicrobial samples, such as those involving sputum, pus, stool, bile, etc., are not suitable for Tm mapping method, because these types of samples contain similar amounts of two or more species of bacteria. Therefore, when attempting to identify bacteria in these sample types using the Tm mapping method, it is necessary to start with bacterial colonies.

The Tm mapping identification requires a measurement error of no more than 0.1 °C among PCR tubes within the same trial (tube-to-tube variation). As long as an optimal instrument is used, the Tm mapping identification does not depend on a single instrument (Table S4). If the tube-to-tube variation is within ±0.1 °C, then the measurement error in the theoretical Difference Value is within 0.26, whereas the measured values on the blind tests were within 0.28. Considering the range of Difference Values in the blind test, all test isolates with a Difference Value less than or equal to 0.28 have the same possibility of being the bacterial isolate. Even if the results cannot be narrowed down to one bacterial species, the method provides information regarding the genus. This sometimes happens within the genus *Staphylococcus*, because bacteria within this genus have similar Tm mapping shapes (Table S3). Tm similarity does not always interfere with the Tm mapping identification as the Difference Value is determined by each of the seven Tm values (Fig. S4a, b). For example, *Bacteroides dorei*, which is similar to *Bacteroides vulgatus* shown in Table S3, does not always interfere with the identification of *Bacteroides vulgatus* and vice versa (Table S9). There are exceptional occasions when two different identification results with the same Difference Value are obtained, and so cannot be narrowed down to one bacterial species (Fig. S4c). This rarely happens, although it did occur in one experiment (Table S8). All of the problems described above are due to the measurement errors caused by the instrument. If these problems occur, it is better to measure the Tm values again, which only takes another 10 minutes.

To increase the reliability of the Tm mapping method, it is important to minimize tube-to-tube variation of the instruments, and expand the Tm mapping database. Because smaller tube-to-tube variation means even more accurate Tm mapping identification, we look forward to the emergence of new instruments with more equalized tube-to-tube variation. The Tm mapping database is scalable and can be easily modified and updated. Currently, 107 bacterial species are registered in the database. Because the reliability of the Tm mapping method improves with the number of species in the database, we are planning to expand the database with American Type Culture Collection (ATCC) reference strains.

When identifying bacterial isolates, significant differences in the pH and salt concentration of the PCR buffer affect the Tm mapping shape. However, in individual clinical samples, foreign substances and differences in the pH and salt concentration do not affect the Tm mapping identification to any degree. This is because the DNA extraction step equalizes these differences, and the PCR product is diluted 500-fold with molecular-grade distilled water before the second (nested) PCR. In order for everyone to use the same Tm mapping database, the pH and salt concentration of the PCR buffer, the bacterial universal primers and the eukaryote-made thermostable DNA polymerase should be consistent. Therefore, it is better to make a reagent kit.

We would like to emphasize that the Tm mapping method does not involve a high-resolution melting-curve (HRM) analysis. Rapid identification methods using HRM analyses have been previously reported^20^,^21^. HRM analyses primarily rely on differences in the melting curve shapes. The Tm mapping method does not use melting curve shapes, but rather only Tm values. For this reason, although melting curve shapes are affected by various DNA concentrations^21^, Tm values are not (Fig. S5).

Chakravorty and colleagues reported the rapid identification of bacterial isolates using a sloppy molecular beacon (SMB) melting temperature signature technique^22^. In that study, the authors used six Tm values as signatures of bacterial isolates and attempted to identify bacterial isolates using the D value as the distance between two points in six-dimensional space. The basic concept of the SMB melting temperature signature technique is similar to that of the Tm mapping method. In our case, we independently developed our method (a Japanese patent was applied for in 2006 and granted in 2010 and an international patent was applied for in 2007 and granted in 2012). The major difference between these techniques is that the other authors use sloppy molecular beacon, a type of DNA probe, while we use bacterial universal PCR primers. Therefore, Tm variations in the SMB melting temperature signature technique depend on the number and position of probe-target mismatches on sloppy molecular beacon hybridization. In contrast, the Tm variations observed in the Tm mapping method depend on the base sequence differences of the amplicons. Therefore, the SMB melting temperature signature technique can be used to generate a wider range of Tm values than the Tm mapping method. However, the wider range of Tm values does not mean that this method is able to identify a wider range of bacterial isolates than the Tm mapping method, although it does mean that tube-to-tube variation is not a major problem for the SMB melting temperature signature technique. Regarding sensitivity, the sloppy molecular beacon is not a PCR primer; hence, bacterial DNA cannot be amplified with it. Furthermore, the sensitivity of the SMB melting temperature signature technique is dependent on linear-after-the-exponential (LATE)-PCR, indicating that it may be difficult to identify bacterial isolates directly from patient samples. In the above report, the SMB melting temperature signature technique was validated in cultured clinical isolates and positive patient blood cultures, not whole blood samples. Meanwhile, the Tm mapping method performs nested PCR using bacterial universal primers and the eukaryote-made thermostable DNA polymerase, thereby making it possible for PCR to identify bacterial isolates directly from patient samples within three hours of sample collection.

The limit of identification is not the same as the limit of detection (LOD) in the Tm mapping method. The LOD is determined using the most sensitive primer among the seven primer sets, and depending on this primer, the absence of bacteria in a sample can be diagnosed. The LOD values for the Tm mapping method are as follows (Table S6): *Escherichia coli* = 0.625 CFU/PCR tube (31.3 CFU/mL), *Staphylococcus aureus* = 0.625 CFU/PCR tube (31.3 CFU/mL), and *Klebsiella pneumoniae* = 0.313 CFU/PCR tube (15.7 CFU/mL). This result indicates that the number of CFU could be lower than the actual number of bacteria in a sample (Table S10). In the current protocol, bacterial DNA is eluted with 100 μL of elution buffer, and 2 μL of which is used as a PCR template. A larger amount of PCR template can be used to achieve higher sensitivity; however, using the current protocol, the Tm mapping method can be employed to identify bacterial isolates directly from whole blood samples. In fact, we quantified the approximate bacterial concentration in the blood of patients with sepsis, and confirmed that the limit of identification of the Tm mapping method is sufficient to identify bacterial isolates directly from whole blood samples (Table S11).

The number of Tm mapping and/or culture positive whole blood samples observed in this study was high because of the inclusion of samples from a geriatric hospital. Among the 200 whole blood samples from patients with suspected sepsis 23% (45/200) were positive according to the conventional culture method and 35% (70/200) were positive according to the Tm mapping method (Table 3). In the geriatric hospital, almost all patients were bedridden, and the Tm mapping method detected bacteria in nearly 50% of the patients with suspected sepsis. The elderly are predisposed to sepsis due to the presence of co-existing co-morbidities, repeated and prolonged hospitalization, reduced immunity, functional limitations and, above all, the effects of aging itself^23^. Of the samples collected from the university hospital, the rate of positivity was around 10%, which is in the normal range. Meanwhile, 70 samples were positive according to the Tm mapping method, of which 39% (27/70) were negative based on the culture method. In general, PCR detects more than cultures because it can also detect dead bacteria. The inconsistent results are primarily the result of the fact that a certain number of patients were already being treated with antibiotics when we collected the samples, which could have resulting in the death of the bacterial cells while retaining detectable DNA. The efficacy of pathogen identification using the blood culture technique is significantly limited by the effects of previously initiated antimicrobial therapy^24^,^25^,^26^. There is also the possibility that some species of oral bacteria or intestinal bacteria, which cannot be identified according to the blood culture technique, may sometimes be circulating in the bloodstream. In order to verify this assumption, it is necessary to perform Tm mapping identification using many healthy blood samples.

In this study, we developed a bacterial contamination-free PCR system. Because we use the eukaryote-made thermostable DNA polymerase, the Tm mapping method can also be applied to diagnose the absence (less than the LOD: e.g. 31.3 CFU/mL of *E. coli*) of bacteria in patient samples within three hours. Of the 130 whole blood samples negative according to the Tm mapping method, two samples were positive based on the culture method (Table 3). However, these bacteria were *Staphylococcus epidermidis* and *Bacillus cereus*, which are often detected as sources of contamination. Therefore, we supposed that the samples had been contaminated during the culture process. It is very important to rapidly distinguish bacterial causes from non-bacterial causes in various clinical conditions, including meningitis, collagen diseases, fever of unknown origin (FUO), etc., in order to choose a suitable treatment. Commercial thermostable DNA polymerases are known to have contamination with host-derived bacterial DNA. When using bacterial universal primers for PCR detection, the contaminating bacterial amplicons can be observed by the 40th cycles of PCR amplification^15^. We devised a nested PCR assay (first PCR: 40 cycles → dilute 500-fold → second, nested PCR: 30 cycles) in which, in approximately 90% of positive samples, amplification was observed not on the first PCR, but only on the second (nested) PCR. For this reason, without the eukaryote-made thermostable DNA polymerase, it would be difficult to identify the bacterial isolate directly from whole blood samples or diagnose the absence of bacteria in a given sample. In order to enable all researchers to perform bacterial contamination-free PCR, we are working to make this eukaryote-made thermostable DNA polymerase commercially available in the near future.

In conclusion, the Tm mapping method enables the identification of the dominant bacteria in a clinical sample within three hours of whole blood collection. In particular, using the Difference Value, it is possible to quickly identify samples suitable for Tm mapping identification. Moreover, this method can be used to rapidly diagnose the absence of bacteria in clinical samples. The Tm mapping method is especially useful for detecting infectious diseases, such as sepsis, that require prompt treatment, and is expected to contribute to the treatment of patients with severe infections as well as reduce the rate of development of antibiotic resistance.

## Methods

### Clinical specimens

A total of 200 whole blood samples were randomly collected from patients with suspected sepsis at Toyama University Hospital and Nagaresugi Geriatric Hospital. All procedures were performed under a protocol approved by the Ethics Committee at the University of Toyama and Nagaresugi Geriatric Hospital, and written informed consent was obtained from all patients. The methods were carried out in accordance with the approved guidelines.

### Isolation of bacterial genomic DNA from whole blood

A total of 2 mL of venous blood or, as a negative control for DNA extraction, 2 mL of molecular-grade distilled water (water deionized and sterilized for molecular biology, NACALAI TESQUE, INC. Kyoto) were collected in EDTA-2K tubes (BD Biosciences Japan, Tokyo, Japan). The blood samples were then centrifuged at 100 × g for five minutes to spin down the blood cells, and the resulting supernatant fractions (1 mL) were used. The supernatants were centrifuged again at 20,000 × g for 10 minutes, and 950 μL of the supernatant fractions was carefully removed in order to not disturb the pellets. Next, 1 mL of molecular-grade distilled water (water deionized and sterilized for molecular biology, NACALAI TESQUE, INC.) was added to the pellets, and the mixture was gently turned upside down several times, and subsequently centrifuged at 20,000 × g for 5 minutes. Finally 1 mL of the supernatant fractions was again carefully removed unless you resuspend the pellet in this before using the DNA extraction kit. DNA was isolated from the pellets using a DNA extraction kit (High Pure PCR Template Preparation Kit, Roche Applied Science, Germany) in accordance with the supplier’s instructions. Finally, bacterial DNA was eluted with 100 μL of elution buffer.

### Isolation of bacterial genomic DNA from colonies

The bacterial colonies were picked up with a sterile inoculating loop and suspended in 1 mL of molecular-grade distilled water (water deionized and sterilized for molecular biology, NACALAI TESQUE, INC.). The samples were subsequently centrifuged at 20,000 × g for 10 minutes, and 950 μL of the supernatant was carefully removed in order to not lose the pellets. DNA was isolated from the resulting pellets using a DNA extraction kit (High Pure PCR Template Preparation Kit, Roche Applied Science) in accordance with the supplier’s instructions. Finally, bacterial DNA was eluted with 100 μL of elution buffer.

### PCR assays

In each of the following processes, the QIAgility system (Qiagen) provided an automated PCR setup. The Rotor-Gene Q (Qiagen) or LightCycler® Nano (Roche Applied Science) was used for the amplification, real-time detection of the target DNA and the Tm value analysis of the amplified products. In particular, when using the LightCycler® Nano, which has two independent thermal blocks, we recommend using the same thermal block for all seven PCR tubes for Tm mapping identification. All PCR assays were performed as single-tube assays (no multiplex PCR). We used 1.5-mL PCR-clean Eppendorf tubes that were RNase- and DNase-free (Eppendorf, Germany), 0.2-mL PCR tubes (Qiagen) for the first PCR and 0.1-mL Strip Tubes and Caps (Qiagen) for the second (nested) PCR. All oligonucleotide primers were designed using a multiple alignment software program (ClustalX) and were synthesized by Life Technologies Japan, Ltd. (Tokyo, Japan). Bacterial universal primers were designed to universally amplify the seven regions of the bacterial 16S ribosomal RNA gene (16S rDNA) (Fig. 2A). The primers were as follows: Region 1 primers (forward: 5′-AGAGTTTGATCATGGCTCAG-3′, reverse: 5′-CGTAGGAGTCTGGACCGT-3′, amplicon size: 338 bp), Region 2 primers (forward: 5′-GACTCCTACGGGAGGCA-3′, reverse: 5′-TATTACCGCGGCTGCTG-3′, amplicon size: 199 bp), Region 3 primers (forward: 5′-AGCAGCCGCGGTAATA-3′, reverse: 5′-GGACTACCAGGGTATCTAATCCT-3′, amplicon size: 287 bp), Region 4 primers (forward: 5′-AACAGGATTAGATACCCTGGTAG-3′, reverse: 5′-AATTAAACCACATGCTCCACC-3′, amplicon size: 181 bp), Region 5 primers (forward: 5′-TGGTTTAATTCGATGCAACGC-3′, reverse: 5′-GAGCTGACGACAGCCAT-3′, amplicon size: 120 bp), Region 6 primers (forward: 5′-TTGGGTTAAGTCCCGC-3′, reverse: 5′-CGTCATCCCCACCTTC-3′, amplicon size: 109 bp), Region 7 primers (forward: 5′-GGCTACACACGTGCTACAAT-3′, reverse: 5′-CCGGGAACGTATTCACC-3′, amplicon size: 166 bp). The first PCR primer set was the same as the Region 1 forward primer and the Region 7 reverse primer (Fig. 2A).

During the first PCR procedure, the PCR reaction mixture (20 μL) contained 2 μL of DNA template in 200 μM of each dNTP (CleanAmp^TM^ Hot Start dNTP Mix, Sigma-Aldrich, USA) filtered using an Amicon Ultra 50 K centrifugal filter (Merck Millipore, Germany), 50 mM KCl, 2.25 mM MgCl_2_, 10 mM Tris-HCl (pH 8.3), 0.3 μM of each primer, 1 × EvaGreen (Biotium Inc. CA, USA), and 1.0 units (0.5 μL) of eukaryote-made thermostable DNA polymerase supplemented with stock buffer solution. The generation of eukaryote-made thermostable DNA polymerase using *Saccharomyces cerevisiae* has been described previously^15^. In place of 2 μL of DNA template, the PCR reaction mixture contained 2 μL (8.0 ng/μL) of DNA extracted from *Escherichia coli* (ATCC 25922) as a positive control, or 2 μL of molecular-grade distilled water (water deionized and sterilized for molecular biology, NACALAI TESQUE, INC.) as a negative control for the PCR step.

Each sample was incubated for five minutes at 95 °C to activate the Hot Start dNTPs, then was denatured for 10 seconds at 94 °C, annealed for 10 seconds at 57 °C, extended for 30 seconds at 72 °C and subjected to fluorescence acquisition for 2 seconds at 82 °C for 40 cycles. The PCR product was diluted 500-fold with molecular-grade distilled water (water deionized and sterilized for molecular biology, NACALAI TESQUE, INC.) and then used as a template for the second (nested) PCR procedure.

For the second (nested) PCR procedure, the PCR reaction mixture (20 μL) contained 2 μL of DNA template of the diluted first PCR product in 200 μM of each dNTP (CleanAmp^TM^ Hot Start dNTP Mix, SIGMA-ALDORICH) filtered using an Amicon Ultra 50 K centrifugal filter (Merck Millipore), 50 mM KCl, 2.5 mM MgCl_2_, 10 mM Tris-HCl (pH 8.3), 0.25 μM of each primer, 1 × EvaGreen (Biotium, Inc.) and 1.0 units (0.5 μL) of eukaryote-made thermostable DNA polymerase supplemented with stock buffer solution. The seven samples used to amplify Regions 1 to 7 were incubated for five minutes at 95 °C to activate the Hot Start dNTPs, then denatured for 10 seconds at 94 °C, annealed for 10 seconds at 57 °C, extended for 10 seconds at 72 °C and subjected to fluorescence acquisition for 2 seconds at 82 °C for 30 cycles. The seven PCR amplicons were then analyzed to obtain the Tm values. If no amplification was observed by the 30th cycle of all 7 secondary PCRs, we defined the sample as containing no bacteria.

### Melting temperature (Tm) value analysis

For the Tm value analysis, the resulting seven amplicons were heated at 95 °C for 10 seconds and then cooled at 72 °C for 90 seconds. A post-PCR Tm value analysis was performed from 72 °C to 95 °C, increasing at 0.5 °C/step. The data profile was subsequently analyzed using the Rotor-Gene Q or LightCycler® Nano software program, and the Tm values were identified.

### Analytical sensitivity tests

Prior to performing the sensitivity tests, *Escherichia coli* was cultivated in Mueller-Hinton Broth at 37 °C for 12 hours, and bacterial suspensions (1 mL) was prepared. 50 μL of the diluted suspensions were inoculated on Standard Methods Agar/Plate Count Agar (BD, USA). After incubation at 35 °C for 10 hours, the number of colony-forming units (CFU) was determined by counting the colonies grown on the agar plates in triplicate.

The limits of identification and detection were determined by serially diluting (log_2_-fold) cultures with known counts (CFU) of *E. coli* in PBS and subjecting the samples to Tm mapping identification or PCR detection using Region 1 to 7 primers. The limit of identification was determined to be the final log_2_ dilution of the template in which the Tm mapping result was correct, with the correct number of PCR amplicons (Table S1) and a Difference Value less than or equal to 0.5. The LOD was determined to be the final log_2_ dilution of the template in which at least one of the seven amplifications was observed by the 30^th^ cycle in the second (nested) PCR.

### Nucleotide sequence-based analysis of bacterial genomic DNA

Amplicons from the samples used in the first PCR procedure were purified (QIAquick PCR Purification Kit; QIAGEN) and then sequenced (3500 Genetic Analyzer; Applied Biosystems) using the Region 1 forward primer and the Region 6 or 5 reverse primer. The Region 6 reverse primer does not bind to the target region of some species of bacteria (Table S1). In these cases, we use the Region 5 reverse primer. An online homology search was performed for strain identification using the BLAST nucleotide database tool of the DNA Data Bank of Japan (http://www.ddbj.nig.ac.jp/index-j.html). The presence of several species of bacteria in a sample was confirmed based on overlapping sequence data (reads). This sequencing method can be used to identify the dominant bacteria in a sample; however, when the sample contains similar amounts of two or more species of bacteria, the sequencing method cannot identify the bacterial isolate due to the presence of multiple overlapping reads.

### Culture-based biochemical identification of bacteria

The whole blood samples (one aerobic blood culture bottle and one anaerobic blood culture bottle, respectively) were collected simultaneously with the blood sample for Tm analysis from the same puncture site. The whole blood samples were then analyzed according to standard methods used by the Clinical Laboratory Center (certified ISO15189) at Toyama University Hospital. The blood culture procedures were performed using the BacT/ALERT 3D system (bioMerieux, Inc., Mercy-l’Etoile, France). Positive blood culture bottles were subcultured in the appropriate media and incubated aerobically or anaerobically until sufficient growth was present to proceed with testing (usually 18 to 24 hours). The specific identification methods differed according to the organism, although they included the MicroScan WalkAway system (Siemens Healthcare Diagnostics, IL, USA), RapID ANA II (Thermo Fisher SCIENTICIC, UK) and various latex agglutination and biochemical spot tests.

## Additional Information

**How to cite this article**: Niimi, H. *et al.* Melting Temperature Mapping Method: A Novel Method for Rapid Identification of Unknown Pathogenic Microorganisms within Three Hours of Sample Collection. *Sci. Rep.* **5**, 12543; doi: 10.1038/srep12543 (2015).

## Supplementary Material

Supplementary Information

## Figures and Tables

**Table 1:** Interpretative criteria.

Difference Value (D)	Suitability for identification	Identification	Interpretation
0.0 ≤ D ≤ 0.28	High	All identification results within this range have the same possibility of being the bacterial isolate	Matched the bacteria registered in the database
0.28 < D ≤ 0.5	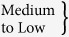	The identification result with the lowest Difference Value is highly likely to be the bacterial isolate	Mutant strain, or Polymicrobial infection*1
0.5 < D	Not suitable	Does NOT identify the bacterial isolate	Polymicrobial infection*2, or Not registered in the database, or Under the limit of identification*3

^*1^Polymicrobial infection with one dominant bacterial species.

^*2^Polymicrobial infection with no dominant bacterial species.

^*3^Under the limit of identification, but over or equal to the limit of detection.

**Table 2:** Comparison of the Tm mapping and culture/sequencing results starting from bacterial colonies.

Difference Value (D	No. of samples	vs. Conventional culture method			vs. Sequencing method		
		No. of matches	No. of broad matches	No. of mismatches	No. of matches	No. of broad matches	No. of mismatches
0.0 ≤ D ≤ 0.28	108	102 (2^a^)	2	4	106	2	0
0.28 < D ≤ 0.5	22	16	1	5	19	1	2
0.5 < D^b^	10	6	0	4	6	0	4

^a^The number of matches at the genus level. In these cases, bacterial isolates were identified using the conventional culture method at the genus level, not the species level.

^b^If the Difference Value is greater than 0.5, the result is below the level of identification and is not suitable for Tm mapping identification. Details of the identification results for these samples is only included here to demonstrate the accuracy of the Tm mapping method.

**Table 3:** Comparison of the Tm mapping and culture results starting from whole blood samples.

Bacterial isolates	Tm mapping method			
	detection	+	−	Total
**Conventional culture method**	+	43^A^(I: 41, NS: 2)	2^C^	45
	−	27^B^(I: 18, NS: 9)	128^D^	155
	Total	70(I: 59, NS: 11)	130	200

**I**: Identified according to the Tm mapping method (Difference Value ≤0.5).

**NS**: Bacteria were detected, but not suitable for Tm mapping identification (Difference Value >0.5).

^A^41 Tm mapping identifications matched the culture (or sequencing) results, whereas two samples were not suitable for Tm mapping identification due to the presence of polymicrobial infection.

^B^18 species could be identified and nine samples were not suitable for Tm mapping identification.

^C^These two samples were positive for *Staphylococcus epidermidis* and *Bacillus cereus*.

^D^These samples were negative using both the Tm mapping method and culture method.

**Table 4:** Individual results of identification starting from whole blood samples.

	Identification results				Match		Comments
	Tm mapping method	Diff.	Conventional culture method	Sequencing method	Cult.	Seq.	
Control: Healthy whole blood							
	None detected	**—**	No culture growth		**—**	**—**	Negative control
Patient							
1	*Staphylococcus capitis/epidermidis*	0.09	*Staphylococcus epidermidis*		✓	**—**	
2	*Staphylococcus capitis/epidermidis*	0.12	*Staphylococcus epidermidis*		✓	**—**	
3	*Staphylococcus capitis/epidermidis*	0.13	No culture growth	*Staphylococcus epidermidis*	**—**	✓	
4	*Streptococcus agalactiae*	0.13	*Streptococcus agalactiae Escherichia coli*	*Streptococcus agalactiae*	✓	✓	dominant bacteria
5	*Staphylococcus aureus*	0.14	*Staphylococcus aureus*		✓	**—**	
6	*Staphylococcus capitis/epidermidis*	0.15	*Staphylococcus epidermidis*		✓	**—**	
7	*Staphylococcus haemolyticus*	0.15	*Staphylococcus haemolyticus*		✓	**—**	
8	*Bacteroides vulgatus*	0.15	*Bacteroides vulgatus*		✓	**—**	
9	*Acinetobacter baumannii*	0.16	No culture growth	*Acinetobacter baumannii*	**—**	✓	
10	*Staphylococcus aureus*	0.16	*Staphylococcus aureus*		✓	**—**	
11	*Escherichia coli*	0.16	No culture growth	*Escherichia coli*	**—**	✓	
12	*Escherichia coli*	0.16	*Escherichia coli*		✓	**—**	
13	*Bacillus cereus*	0.17	No culture growth	*Bacillus cereus*	**—**	✓	
14	*Staphylococcus haemolyticus*	0.17	*Staphylococcus haemolyticus*		✓	**—**	
15	*Escherichia coli*	0.18	No culture growth	*Escherichia coli*	**—**	✓	
16	*Staphylococcus capitis/epidermidis*	0.18	No culture growth	*Staphylococcus epidermidis*	**—**	✓	
17	*Streptococcus pyogenes*	0.18	*Streptococcus sanguinis*	*Streptococcus pyogenes*	×	✓	
18	*Staphylococcus capitis/epidermidis*	0.18	*Staphylococcus epidermidis*		✓	**—**	
19	*Escherichia coli*	0.18	*Escherichia coli*		✓	**—**	
20	*Staphylococcus caprae*	0.18	*Staphylococcus epidermidis*	*Staphylococcus caprae*	×	✓	
21	*Enterococcus facium*	0.18	*Enterococcus facium*		✓	**—**	
22	*Staphylococcus aureus*	0.19	No culture growth	*Staphylococcus aureus*	**—**	✓	
23	*Staphylococcus aureus*	0.19	*Staphylococcus aureus*		✓	**—**	
24	*Staphylococcus capitis/epidermidis*	0.19	*Staphylococcus epidermidis*		✓	**—**	
25	*Staphylococcus caprae*	0.20	*Staphylococcus epidermidis*	*Staphylococcus caprae*	×	✓	
26	*Pseudomonas aeruginosa*	0.20	*Pseudomonas aeruginosa*		✓	**—**	
27	*Bacillus cereus*	0.20	*Bacillus cereus*		✓	**—**	
28	*Staphylococcus haemolyticus*	0.21	*Staphylococcus aureus*	*Staphylococcus haemolyticus*	×	✓	
29	*Escherichia coli*	0.21	*Escherichia coli Bacteroides thetaiotaomicron Bacteroides vulgatus*	*Escherichia coli*	✓	✓	dominant bacteria
30	*Escherichia coli*	0.21	*Escherichia coli*		✓	**—**	
31	*Staphylococcus haemolyticus*	0.22	No culture growth	*Staphylococcus haemolyticus*	**—**	✓	
32	*Staphylococcus capitis/epidermidis*	0.22	*Staphylococcus epidermidis*		✓	**—**	
33	*Staphylococcus caprae*	0.22	*Staphylococcus epidermidis*	*Staphylococcus caprae*	×	✓	
34	*Staphylococcus haemolyticus* or	0.22	*Staphylococcus haemolyticus*	*Staphylococcus haemolyticus*	✓	✓	broad match
	*Staphylococcus lugdunensis*	0.26					
35	*Staphylococcus caprae*	0.24	No culture growth	*Staphylococcus caprae*	**—**	✓	
36	*Staphylococcus aureus*	0.24	No culture growth	*Staphylococcus aureus*	**—**	✓	
37	*Staphylococcus capitis/epidermidis*	0.24	*Staphylococcus epidermidis*		✓	**—**	
38	*Escherichia coli*	0.24	No culture growth	*Escherichia coli*	**—**	✓	
39	*Klebsiella pneumoniae*	0.25	*Klebsiella pneumoniae*		✓	**—**	
40	*Staphylococcus capitis/epidermidis*	0.26	*Staphylococcus epidermidis*		✓	**—**	
41	*Escherichia coli*	0.26	*Escherichia coli*		✓	**—**	
42	*Citrobacter freundii*	0.26	*Citrobacter freundii*		✓	**—**	
43	*Staphylococcus caprae*	0.27	*Staphylococcus epidermidis*	*Staphylococcus caprae*	×	✓	
44	*Acinetobacter calcoaceticus*	0.28	No culture growth	*Acinetobacter calcoaceticus*	**—**	✓	
45	*Escherichia coli*	0.28	*Escherichia coli Enterococcus faecalis*	*Escherichia coli*	✓	✓	dominant bacteria
46	*Staphylococcus hominis*	0.28	*Staphylococcus hominis*		✓	**—**	
47	*Escherichia coli*	0.30	No culture growth	*Escherichia coli*	**—**	✓	
48	*Escherichia coli*	0.30	*Escherichia coli*		✓	**—**	
49	*Klebsiella pneumoniae*	0.30	*Klebsiella pneumoniae*		✓	**—**	
50	*Staphylococcus lugdunensis*	0.31	No culture growth	*Staphylococcus lugdunensis*	**—**	✓	
51	*Klebsiella pneumoniae*	0.31	*Klebsiella pneumoniae*		✓	**—**	
52	*Staphylococcus capitis/epidermidis*	0.32	*Staphylococcus epidermidis*		✓	**—**	
53	*Staphylococcus warneri*	0.33	No culture growth	*Staphylococcus warneri*	**—**	✓	
54	*Enterobacter cloacae*	0.35	*Enterobacter cloacae*		✓	**—**	
55	*Streptococcus pyogenes*	0.36	No culture growth	*Streptococcus pyogenes*	**—**	✓	
56	*Escherichia coli*	0.38	*Escherichia coli*		✓	**—**	
57	*Staphylococcus hominis*	0.39	No culture growth	*Staphylococcus hominis*	**—**	✓	
58	*Staphylococcus cohnii*	0.43	No culture growth	*Staphylococcus cohnii*	**—**	✓	
59	*Aeromonas hydrophila*	0.50	*Aeromonas hydrophila*	*Aeromonas hydrophila*	✓	✓	

Diff. = Difference Value, Cult. = Culture results, Seq. = Sequencing results, DB = Database.

✓: matched the culture/sequencing result.

×: did not match the culture/sequencing result.

—: did not perform a comparison with the culture/sequencing result.

The bold line in the Difference Value column marks the interpretative criteria boundary (0.28) described in Table 1.
