# Supplementary material for: Melting Temperature Mapping Method: A Novel Method for Rapid Identification of Unknown Pathogenic Microorganisms within Three Hours of Sample Collection
Source: Sci Rep. 2015 Jul 28;5:12543. doi: 10.1038/srep12543 (PMC4517391; doi:10.1038/srep12543)
Supplement: Supplementary Information [file srep12543-s1.pdf]

Supplementary Information

Melting Temperature Mapping Method: A Novel Method for Rapid Identification of Unknown Pathogenic Microorganisms within Three Hours of Sample Collection

Hideki Niimi[1*], Tomohiro Ueno[1], Shirou Hayashi[1], Akihito Abe[2], Takahiro Tsurue[2], Masashi Mori[3], Homare Tabata[4], Hiroshi Minami[4], Michihiko Goto[5], Makoto Akiyama[6], Yoshihiro Yamamoto[7], Shigeru Saito[8] and Isao Kitajima[1*]

[1]Clinical Laboratory Center, Toyama University Hospital, Toyama 930-0194, Japan, [2]Kitami Information Technology Co., Ltd., Hokkaido 090-0813, Japan, [3]Research Institute for Bioresources and Biotechnology, Ishikawa Prefectural University, Ishikawa 921-8836, Japan, [4]Life Science Center, Hokkaido Mitsui Chemicals, Inc., Hokkaido 073-0138, Japan, [5]Department of Internal Medicine, University of Iowa Carver College of Medicine, IA 52242, USA, and Iowa City Veterans Affairs Medical Center, IA 52246, USA, [6]Nagaresugi Geriatric Hospital, Toyama 939-8032, Japan, [7]Department of Clinical Infectious Diseases, Toyama University Hospital, Toyama 930-0194, Japan, [8]Department of Obstetrics & Gynecology, Toyama University Hospital, Toyama 930-0194, Japan

*Correspondence to:

Hideki Niimi, M.D. Ph.D.   E-mail address: hiniimi@med.u-toyama.ac.jp

Isao Kitajima, M.D. Ph.D.   E-mail address: kitajima@med.u-toyama.ac.jp

Clinical Laboratory Center, Toyama University Hospital, 2630 Sugitani, Toyama 930-0194, JAPAN

Phone: (+81)-76-434-7759, Fax: (+81)-76-434-7759

Supplemental Table S1

The seven universal primers binding to the bacterial target regions registered in the database

Bacteria in the database	Primer bindings						
	Reg. 1	Reg. 2	Reg. 3	Reg. 4	Reg. 5	Reg. 6	Reg. 7
Achromobacter xylosoxidans	+	+	+	+	+	+	+
Acinetobacter baumannii	+	+	+	+	+	+	+
Acinetobacter calcoaceticus	+	+	+	+	+	+	+
Aerococcus christensenii	+	+	+	+	+	+	+
Aeromonas hydrophilia	+	+	+	+	+	+	+
Alistipes onderdonkii	+	+	+	+	+	−	+
Anaeroglobus geminatus	+	+	+	+	+	+	+
Arthrobacter cumminsii	+	+	+	+	+	+	+
Atopobium vaginae	+	+	+	+	+	+	+
Bacillus cereus	+	+	+	+	+	+	+
Bacteroides dorei	+	+	+	+	+	−	+
Bacteroides finegoldii	+	+	+	+	+	−	+
Bacteroides fragilis	+	+	+	+	+	−	+
Bacteroides nordii	+	+	+	+	+	−	+
Bacteroides salyersiae	+	+	+	+	+	−	+
Bacteroides thetaiotaomicron	+	+	+	+	+	−	+
Bacteroides uniformis	+	+	+	+	+	−	+
Bacteroides vulgatus	+	+	+	+	+	−	+
Bifidobacterium bifidum	+	+	+	+	+	+	+
Bifidobacterium breve	+	+	+	+	+	+	+
Bilophila wadsworthia	+	+	+	+	+	+	+
Campylobacter curvus	+	+	+	+	+	+	+
Campylobacter jejuni	+	+	+	+	+	+	+
Campylobacter rectus	+	+	+	+	+	+	+
Citrobacter freundii	+	+	+	+	+	+	+
Clostridium difficile	+	+	+	+	+	+	+
Clostridium hylemonae	+	+	+	+	+	+	+
Clostridium perfringens	+	+	+	+	+	+	+
Clostridium tertium	+	+	+	+	+	+	+
Corynebacterium amycolatum	+	+	+	+	+	+	+
Corynebacterium macginleyi	+	+	+	+	+	+	+
Corynebacterium xerosis	+	+	+	+	+	+	+
Eggerthella lenta	+	+	+	+	+	+	+
Eikenella corrodens	+	+	+	+	+	+	+
Enterobacter aerogenes	+	+	+	+	+	+	+
Enterobacter cloacae	+	+	+	+	+	+	+
Enterococcus avium	+	+	+	+	+	+	+
Enterococcus casseliflavus	+	+	+	+	+	+	+
Enterococcus faecalis	+	+	+	+	+	+	+
Enterococcus faecium	+	+	+	+	+	+	+
Escherichia albertii	+	+	+	+	+	+	+
Escherichia coli	+	+	+	+	+	+	+
Eubacterium lentum	+	+	+	−	+	+	+
Eubacterium limosum	+	+	+	+	+	+	+
Finegoldia magna	+	+	+	+	+	+	+
Fusobacterium nucleatum	+	+	+	+	+	+	+
Fusobacterium periodonticum	+	+	+	+	+	+	+
Gardnerella vaginalis	+	+	+	+	+	+	+
Gemella morbillorum	+	+	+	+	+	+	+
Granulicatella adiacens	+	+	+	+	+	+	+
Haemophilus influenzae	+	+	+	+	+	+	+
Halomonas venusta	+	+	+	+	+	+	+
Klebsiella oxytoca	+	+	+	+	+	+	+
Klebsiella pneumoniae	+	+	+	+	+	+	+
Listeria monocytogenes	+	+	+	+	+	+	+
Lactobacillus crispatus	+	+	+	+	+	+	+
Lactobacillus jensenii	+	+	+	+	+	+	+
Lactococcus garvieae	+	+	+	+	+	+	+

Bacteria in the database	Primer bindings						
	Reg. 1	Reg. 2	Reg. 3	Reg. 4	Reg. 5	Reg. 6	Reg. 7
Morganella morganii	+	+	+	+	+	+	+
Mycoplasma genitalium	+	+	+	+	–	+	+
Mycoplasma hominis	+	+	+	+	–	–	+
Odoribacter splanchnicus	+	+	+	+	+	–	+
Pantoea agglomerans	+	+	+	+	+	+	+
Parabacteroides distasonis	+	+	+	+	+	–	+
Parvimonas micra	+	+	+	+	+	+	+
Pasteurella multocida	+	+	+	+	+	+	+
Peptoniphilus gorbachii	+	+	+	–	+	+	+
Peptostreptococcus anaerobius	+	+	+	+	+	+	+
Porphyromonas gingivalis	+	+	+	+	+	–	+
Prevotella bivia	+	+	+	+	+	–	+
Prevotella intermedia	+	+	+	+	+	–	+
Prevotella melaninogenica	+	+	+	+	+	–	+
Prevotella nigrescens	+	+	+	+	+	–	+
Propionibacterium acnes	+	+	+	–	+	+	+
Propioniabacterium avidum	+	+	+	+	+	+	+
Proteus mirabilis	+	+	+	+	+	+	+
Proteus vulgaris	+	+	+	+	+	+	+
Providencia rettgeri	+	+	+	+	+	+	+
Pseudomonas aeruginosa	+	+	+	+	+	+	+
Pseudomonas putida	+	+	+	+	+	+	+
Salmonella enterica	+	+	+	+	+	+	+
Serratia marcescens	+	+	+	+	+	+	+
Staphylococcus aureus	+	+	+	+	+	+	+
Staphylococcus capitis/epidermidis	+	+	+	+	+	+	+
Staphylococcus caprae	+	+	+	+	+	+	+
Staphylococcus cohnii	+	+	+	+	+	+	+
Staphylococcus haemolyticus	+	+	+	+	+	+	+
Staphylococcus hominis	+	+	+	+	+	+	+
Staphylococcus lugdunensis	+	+	+	+	+	+	+
Staphylococcus pettenkoferi	+	+	+	+	+	+	+
Staphylococcus schleiferi	+	+	+	+	+	+	+
Staphylococcus warneri	+	+	+	+	+	+	+
Stenotrophomonas maltophilia	+	+	+	+	+	+	+
Streptococcus agalactiae	+	+	+	+	+	+	+
Streptococcus anginosus	+	+	+	+	+	+	+
Streptococcus constellatus	+	+	+	+	+	+	+
Streptococcus dysgalactiae	+	+	+	+	+	+	+
Streptococcus mitis	+	+	+	+	+	+	+
Streptococcus pasteurianus	+	+	+	+	+	+	+
Streptococcus pneumoniae	+	+	+	+	+	+	+
Streptococcus pyogenes	+	+	+	+	+	+	+
Streptococcus salivarius	+	+	+	+	+	+	+
Streptococcus sanguinis	+	+	+	+	+	+	+
Sutterella wadsworthensis	+	+	+	–	+	+	+
Ureaplasma parvum	+	+	+	+	–	+	+
Vagococcus fluvialis	+	+	+	+	+	+	+
Veillonella atypica	+	+	+	+	+	+	+

+: Primers bind to the bacterial target region

–: Primers do not bind or bind very weakly to the bacterial target region

Reg. 1 – 7 = Region 1 – 7 primers

Supplemental Table S2

Low sequence homology between the universal primers and bacterial target regions associated with the unbound primers shown in Table S2

	Forward (5'→3')	Reverse (5'→3')	Difference (bp)	
**Region 4 primers**	AACAGGATTAGATACCCTGGTAG	AATTAAACCACATGCTCCACC	Forward	Reverse
Eubacterium lentum	AACAGGATTAGATACCCTGGTAG	AATTAAGCCACATGCTCCGCT	0	**3**
Peptoniphilus gorbachii	AACAGGATTAGATACCCTGGTAG	AATTAAACCACATGCTCCGCT	0	**2**
Propionibacterium acnes	AACAGGCTTAGATACCCTGGTAG	AATTAATCCGCATGCTCCGCC	**1**	**3**
Sutterella wadsworthensis	AACAGGATTAGATACCCTGGTAG	AATTAATCCACATCATCCACC	0	**3**
**Region 5 primers**	TGGTTTAATTCGATGCAACGC	GAGCTGACGACAGCCAT	Forward	Reverse
Mycoplasma genitalium	TTGCTTAATTCGACGGTACAC	GAGCTGACGACAACCAT	**6**	**1**
Mycoplasma hominis	TGGTTTAATTTGAAGATACAC	GAGCTGACGACAACCAT	**5**	**1**
Ureaplasma parvum	TTGCTTAATTTGACAATACAC	GAGCTGACGACAACCAT	**8**	**1**
**Region 6 primers**	TTGGGTTAAGTCCCGC	CGTCATCCCCACCTTC	Forward	Reverse
Alistipes onderdonkii	TCGGGTTAAGTCCCAT	CGTCATCCCCACCTTC	**3**	0
Bacteroides dorei	TCGGCTTAAGTGCCAT	CGTCATCCCCACCTTC	**5**	0
Bacteroides finegoldii	TCGGCTTAAGTGCCAT	CGTCATCCCCACCTTC	**5**	0
Bacteroides fragilis	TCGGCTTAACTGCCAT	CGTCATCCCCACCTTC	**6**	0
Bacteroides nordii	TCGGCTTAAGTGCCAT	CGTCATCCCCACCTTC	**5**	0
Bacteroides salyersiae	TCGGCTTAAGTGCCAT	CGTCATCCCCACCTTC	**5**	0
Bacteroides thetaiotaomicron	TCGGCTTAAGTGCCAT	CGTCATCCCCACCTTC	**5**	0
Bacteroides uniformis	TCGGCTTAAGTGCCAT	CGTCATCCCCACCTTC	**5**	0
Bacteroides vulgatus	TCGGCTTAAGTGCCAT	CGTCATCCCCACCTTC	**5**	0
Mycoplasma hominis	TTTGGTCAAGTCCTGC	CGTCATCCCCACCTTC	**3**	0
Odoribacter splanchnicus	TCGGGTTAAGTCCCAT	CGTCATCCCCGCCTTC	**3**	**1**
Parabacteroides distasonis	TCGGCTTAAGTGCCAT	CGTCATCCCCG^{V}CTTC	**5**	**2**
Porphyromonas gingivalis	TCGGCTTAAGTGCCAT	CGTCATCCACACCTTC	**5**	**1**
Prevotella bivia	TCGGCTTAAGTGCCAT	CGTCATCCCCACCTTC	**5**	0
Prevotella intermedia	TCGGCTTAAGTGCCAT	CGTCATCCCCACCTTC	**5**	0
Prevotella melaninogenica	TCGGCTTAAGTGCCAT	CGTCATCCCCACCTTC	**5**	0
Prevotella nigrescens	TCGGCTTAAGTGCCAT	CGTCATCCCCACCTTC	**5**	0

Supplemental Table S3 Tm mapping shape similarity (Difference Value: calculated using the registered Tm values) among the 107 species of bacteria in the database.

D: Difference Value

Bacteria in the database	The number of bacteria					Notes; similar bacteria (D ≤ 0.5)
	0 ≤ D ≤ 0.28	0.28 < D ≤ 0.4	0.4 < D ≤ 0.5	0.5 < D ≤ 1.0	1.0 < D	
Achromobacter xylosoxidans	0	0	0	0	106	
Acinetobacter baumannii	0	0	1	0	105	*A. calcoaceticus* (0.45)
Acinetobacter calcoaceticus	0	0	1	3	102	*A. baumannii* (0.45)
Aerococcus christensenii	0	0	0	0	106	
Aeromonas hydrophilia	0	0	0	2	104	
Alistipes onderdonkii	0	0	0	0	106	
Anaeroglobus geminatus	0	0	0	0	106	
Arthrobacter cumminsii	0	0	0	4	102	
Atopobium vaginae	0	0	0	0	106	
Bacillus cereus	0	0	0	3	103	
Bacteroides dorei *	1	0	0	1	104	*B. vulgatus* (0.26)
Bacteroides finegoldii	0	0	0	2	104	
Bacteroides fragilis	0	0	1	3	102	*B. uniformis* (0.41)
Bacteroides nordii	0	0	0	3	103	
Bacteroides salyersiae	0	0	0	2	104	
Bacteroides thetaiotaomicron	0	0	0	1	105	
Bacteroides uniformis	0	0	1	2	103	*B. fragilis* (0.41)
Bacteroides vulgatus *	1	0	0	2	103	*B. dorei* (0.26)
Bifidobacterium bifidum	0	0	0	0	106	
Bifidobacterium breve	0	0	0	0	106	
Bilophila wadsworthia	0	0	0	0	106	
Campylobacter curvus	0	0	0	0	106	
Campylobacter jejuni	0	0	0	1	105	
Campylobacter rectus	0	0	0	2	104	
Citrobacter freundii	0	0	0	4	102	
Clostridium difficile	0	0	1	0	105	*P. anaerobius* (0.43)
Clostridium hylemonae	0	0	0	0	106	
Clostridium perfringens	0	0	0	1	105	
Clostridium tertium	0	0	0	1	105	
Corynebacterium amycolatum	0	0	0	0	106	
Corynebacterium macginleyi	0	0	0	0	106	
Corynebacterium xerosis	0	0	0	0	106	
Eggerthella lenta	0	0	0	3	103	
Eikenella corrodens	0	0	0	0	106	
Enterobacter aerogenes	0	0	0	2	104	
Enterobacter cloacae	0	1	0	5	100	*P. agglomerans* (0.36)
Enterococcus avium	0	0	1	4	101	*E. faecium* (0.41)
Enterococcus casseliflavus	0	0	0	2	104	
Enterococcus faecalis	0	0	0	2	104	
Enterococcus faecium	0	0	1	3	102	*E. avium* (0.41)
Escherichia albertii	0	0	0	7	99	
Escherichia coli	0	0	0	9	97	
Eubacterium lentum	0	0	0	0	106	
Eubacterium limosum	0	0	0	0	106	
Finegoldia magna	0	0	0	1	105	
Fusobacterium nucleatum	0	0	1	0	105	*F. periodonticum* (0.44)
Fusobacterium periodonticum	0	0	1	1	104	*F. nucleatum* (0.44)
Gardnerella vaginalis	0	0	0	0	106	
Gemella morbillorum	0	0	0	1	105	
Granulicatella adiacens	0	0	0	0	106	
Haemophilus influenzae	0	0	1	8	97	*S. caprae* (0.43)
Halomonas venusta	0	0	0	0	106	
Klebsiella oxytoca	0	0	0	4	102	
Klebsiella pneumoniae	0	0	0	7	99	
Listeria monocytogenes	0	0	0	0	106	
Lactobacillus crispatus	0	0	0	2	104	
Lactobacillus jensenii	0	0	0	3	103	
Lactococcus garvieae	0	0	0	1	105	
Morganella morganii	0	0	0	8	98	
Mycoplasma genitalium	0	0	0	0	106	
Mycoplasma hominis	0	0	0	0	106	
Pantoea agglomerans	0	1	0	4	101	*E. cloacae* (0.36)
Odoribacter splanchnicus	0	0	0	0	106	
Parabacteroides distasonis	0	0	0	1	105	
Parvimonas micra	0	0	0	0	106	

D: Difference Value

Bacteria in the database	The number of bacteria					Notes; similar bacteria (D ≤ 0.5)
	0 ≤ D ≤ 0.28	0.28 < D ≤ 0.4	0.4 < D ≤ 0.5	0.5 < D ≤ 1.0	1.0 < D	
Pasteurella multocida	0	0	0	1	105	
Peptoniphilus gorbachii	0	0	0	0	106	
Peptostreptococcus anaerobius	0	0	1	1	104	*C. difficile* (0.43)
Porphyromonas gingivalis	0	0	0	0	106	
Prevotella bivia	0	0	0	3	103	
Prevotella intermedia	0	0	0	0	106	
Prevotella melaninogenica	0	0	0	3	103	
Prevotella nigrescens	0	0	0	0	106	
Propionibacterium acnes	0	0	0	0	106	
Propioniabacterium avidum	0	0	0	0	106	
Proteus mirabilis	0	0	0	5	101	
Proteus vulgaris	0	0	0	3	103	
Providencia rettgeri	0	0	0	3	103	
Pseudomonas aeruginosa	0	0	0	1	105	
Pseudomonas putida	0	0	0	1	105	
Salmonella enterica	0	0	0	2	104	
Serratia marcescens	0	0	0	11	95	
Staphylococcus aureus	0	1	0	12	93	*S. hemolyticus* (0.35)
Staphylococcus capitis/epidermidis	0	1	0	7	98	*S. pettenkoferi* (0.35)
Staphylococcus caprae	0	0	1	10	95	*H. influenzae* (0.43)
Staphylococcus cohnii	0	0	1	8	97	*S. warneri* (0.43)
Staphylococcus haemolyticus	0	2	1	12	91	*S. lugdunensis* (0.32), *S. aureus* (0.35), *S. warneri* (0.45)
Staphylococcus hominis	0	0	0	6	100	
Staphylococcus lugdunensis	0	1	1	12	92	*S. haemolyticus* (0.32), *S. warneri* (0.43)
Staphylococcus pettenkoferi	0	1	1	8	96	*S. capitis/epidermidis* (0.35)
Staphylococcus schleiferi	0	0	0	1	105	
Staphylococcus warneri	0	0	3	6	97	*S. cohnii* (0.43), *S. lugdunensis* (0.43), *S. haemolyticus* (0.45)
Stenotrophomonas maltophilia	0	0	0	3	103	
Streptococcus agalactiae	0	0	0	3	103	
Streptococcus anginosus	0	0	0	0	106	
Streptococcus constellatus	0	0	0	4	102	
Streptococcus dysgalactiae	0	0	0	2	104	
Streptococcus mitis	0	0	1	5	100	*S. pneumoniae* (0.48)
Streptococcus pasteurianus	0	0	0	2	104	
Streptococcus pneumoniae	0	0	1	3	102	*S. mitis* (0.48)
Streptococcus pyogenes	0	0	0	4	102	
Streptococcus salivarius	0	0	0	1	105	
Streptococcus sanguinis	0	0	0	2	104	
Sutterella wadsworthensis	0	0	0	0	106	
Ureaplasma parvum	0	0	0	0	106	
Vagococcus fluvialis	0	0	0	6	100	
Veillonella atypica	0	0	0	0	106	

This table shows the number of bacterial species registered in the database with a similar shape based on the Difference Value. In other words, this table shows the specificity of each species of bacteria based on mutual Tm mapping shape similarity. Each of the Difference Values was calculated using the Tm values already registered in the database. Bacteria with similar Tm mapping shapes (Difference Value ≤ 0.28) are shown by asterisks. This similarity does not always interfere with the Tm mapping identification, as the Difference Value is determined by each of the seven Tm values (**Fig. S4**). Therefore, *Bacteroides dorei* does not always interfere with the identification of *Bacteroides vulgatus* and vice versa (**Table S9**).

Supplemental Table S4

Validation of the measurement errors among three different instruments and trials using the same *E. coli* (ATCC25922) DNA template

RotorGeneQ (QIAGEN): First instrument

	Triplicate	Reg. 1	Reg. 2	Reg. 3	Reg. 4	Reg. 5	Reg. 6	Reg. 7	Diff.
First Trial	1	89.35	88.60	89.35	89.90	86.25	87.75	87.50	0.21
	2	89.40	88.50	89.40	89.85	86.25	87.75	87.50	0.24
	3	89.35	88.50	89.40	89.90	86.25	87.75	87.50	0.19
Second Trial	1	89.25	88.60	89.40	89.90	86.35	87.75	87.50	0.19
	2	89.40	88.65	89.50	90.00	86.40	87.85	87.60	0.17
	3	89.35	88.65	89.50	90.00	86.35	87.75	87.60	0.13
Third Trial	1	89.35	88.50	89.40	89.85	86.25	87.65	87.40	0.23
	2	89.35	88.50	89.40	89.90	86.25	87.65	87.50	0.19
	3	89.25	88.60	89.50	89.90	86.25	87.75	87.50	0.17

The range of the Difference Values = 0.13 to 0.24

The mean Difference Value = 0.191

RotorGeneQ (QIAGEN): Second instrument

	Triplicate	Reg. 1	Reg. 2	Reg. 3	Reg. 4	Reg. 5	Reg. 6	Reg. 7	Diff.
First Trial	1	89.35	88.60	89.40	89.90	86.35	87.65	87.50	0.21
	2	89.35	88.60	89.50	90.00	86.40	87.65	87.50	0.13
	3	89.40	88.65	89.50	90.00	86.35	87.75	87.60	0.15
Second Trial	1	89.25	88.50	89.40	89.90	86.35	87.65	87.40	0.16
	2	89.25	88.60	89.50	89.90	86.35	87.75	87.50	0.20
	3	89.35	88.60	89.50	89.90	86.35	87.75	87.60	0.15
Third Trial	1	89.25	88.60	89.50	90.00	86.35	87.65	87.50	0.14
	2	89.35	88.65	89.50	90.00	86.40	87.75	87.50	0.19
	3	89.35	88.65	89.50	89.90	86.35	87.75	87.60	0.15

The range of the Difference Values = 0.13 to 0.21

The mean Difference Value = 0.164

LightCycler® Nano (Roche Applied Science)

	Triplicate	Reg. 1	Reg. 2	Reg. 3	Reg. 4	Reg. 5	Reg. 6	Reg. 7	Diff.
**First Trial**	**1**	89.46	88.64	89.48	89.93	86.38	87.75	87.63	0.20
	**2**	89.50	88.62	89.50	89.97	86.38	87.86	87.65	0.21
	**3**	89.47	88.60	89.47	90.02	86.44	87.88	87.73	0.19
**Second Trial**	**1**	89.46	88.64	89.46	89.95	86.33	87.83	87.67	0.17
	**2**	89.51	88.62	89.49	90.01	86.41	87.80	87.63	0.22
	**3**	89.47	88.63	89.47	89.99	86.41	87.81	87.72	0.17
**Third Trial**	**1**	89.38	88.47	89.32	89.69	86.14	87.57	87.54	0.28
	**2**	89.45	88.55	89.47	89.93	86.37	87.70	87.58	0.21
	**3**	89.36	88.57	89.41	89.93	86.34	87.78	87.62	0.14

Range of the Difference Values = 0.14 to 0.28

Mean Difference Value = 0.199

Reg. 1 – 7 = melting temperatures of the amplicons amplified by the Region 1 – 7 primers

Diff. = Difference Value

Supplemental Table S5

Range of Difference Values among 15 different trials starting from DNA extraction of the same *E. coli* (ATCC25922)

RotorGeneQ (QIAGEN)

Trials	Reg. 1	Reg. 2	Reg. 3	Reg. 4	Reg. 5	Reg. 6	Reg. 7	Diff.
**1st**	89.25	88.50	89.40	89.85	86.35	87.75	87.60	0.16
**2nd**	89.50	88.75	89.60	90.00	86.50	87.85	87.75	0.18
**3rd**	89.40	88.65	89.50	90.00	86.40	87.75	87.60	0.17
**4th**	89.25	88.50	89.25	89.85	86.25	87.65	87.50	0.17
**5th**	89.50	88.50	89.50	89.90	86.25	87.75	87.75	0.26
**6th**	89.25	88.50	89.40	89.90	86.25	87.75	87.50	0.15
**7th**	89.25	88.50	89.40	89.85	86.35	87.65	87.50	0.17
**8th**	89.25	88.50	89.35	89.90	86.35	87.75	87.75	0.19
**9th**	89.35	88.65	89.50	90.00	86.40	87.75	87.60	0.13
**10th**	89.25	88.50	89.40	90.00	86.25	87.75	87.50	0.17
**11th**	89.35	88.50	89.35	89.90	86.40	87.75	87.50	0.23
**12th**	89.25	88.50	89.35	89.85	86.25	87.65	87.50	0.13
**13th**	89.25	88.50	89.40	89.90	86.35	87.75	87.50	0.19
**14th**	89.25	88.50	89.50	90.00	86.40	87.75	87.50	0.13
**15th**	89.25	88.50	89.50	89.85	86.50	87.65	87.40	0.25

Range of the Difference Values = 0.13 to 0.26

Mean Difference Value = 0.179

Reg. 1 – 7 = melting temperatures of the amplicons amplified by the Region 1 – 7 primers

Diff. = Difference Value

Supplemental Table S6

Validation of limit of identification and limit of detection (LOD) of the Tm mapping method using the same *E. coli* (ATCC25922) DNA template

a) *Escherichia coli* (three independent experiments were performed in triplicate)

CFU/ PCR Tube	CFU/mL	Tm values							Tm mapping identification results	Difference Value
		Reg. 1	Reg. 2	Reg. 3	Reg. 4	Reg. 5	Reg. 6	Reg. 7		
20.0	1000	89.45	88.70	89.55	90.10	86.20	87.70	87.75	*E. coli*	0.23
		89.50	88.75	89.65	90.10	86.50	87.75	87.65	*E. coli*	0.25
		89.85	89.00	89.90	90.25	86.65	88.00	88.10	*E. coli*	0.26
10.0	500	89.40	88.60	89.50	90.00	86.10	87.65	87.70	*E. coli*	0.24
		89.50	88.85	89.65	90.15	86.40	87.75	87.75	*E. coli*	0.22
		89.80	88.90	89.90	90.35	86.65	88.00	88.15	*E. coli*	0.23
5.00	250	89.40	88.65	89.50	90.00	86.15	87.65	87.60	*E. coli*	0.23
		89.60	88.80	89.75	90.25	86.50	87.85	87.75	*E. coli*	0.23
		89.85	89.00	90.00	90.35	86.65	88.00	88.15	*E. coli*	0.27
2.50	125	89.40	88.70	89.60	90.00	86.25	87.60	87.65	*E. coli*	0.25
		89.65	88.80	89.75	90.15	86.50	87.80	87.80	*E. coli*	0.26
		89.80	88.90	89.90	90.25	86.65	88.00	88.15	*E. coli*	0.25
1.25*1	62.5*1	89.40	88.65	89.50	89.95	86.10	87.50	87.70	*E. coli*	0.33
		89.60	88.70	89.65	90.05	86.40	87.65	87.80	*E. coli*	0.31
		89.85	88.90	89.90	90.25	86.65	87.90	88.15	*E. coli*	0.34
0.625*2	31.3*2	N.D.	N.D.	89.70	90.15	N.D.	N.D.	87.70	Not identified	–
		89.70	N.D.	89.65	N.D.	86.50	N.D.	87.75	Not identified	–
		90.00	N.D.	89.90	N.D.	86.75	N.D.	88.15	Not identified	–
0.313	15.6	N.D.	N.D.	N.D.	N.D.	N.D.	N.D.	N.D.	Not detected	–
		N.D.	N.D.	89.70	N.D.	N.D.	N.D.	87.75	Not identified	–
		N.D.	N.D.	90.00	N.D.	N.D.	N.D.	N.D.	Not identified	–

***1**: limit of identification, ***2**: limit of detection (LOD)

Reg. 1 – 7 = melting temperatures of the amplicons amplified by the Region 1 – 7 primers

CFU = Colony Forming Unit, N.D. = Not detected

The protocol of this validation is described in the methods section under Analytical sensitivity tests.

b) *Staphylococcus aureus* (three independent experiments were performed in triplicate)

CFU/ PCR Tube	CFU/mL	Tm values							Tm mapping identification results	Difference Value
		Reg. 1	Reg. 2	Reg. 3	Reg. 4	Reg. 5	Reg. 6	Reg. 7		
20.0	1000	88.30	87.75	88.40	89.00	85.05	86.50	86.00	*S. aureus*	0.28
		88.25	87.50	88.15	88.75	84.90	86.40	85.75	*S. aureus*	0.21
		88.50	87.65	88.60	89.00	85.50	86.60	86.15	*S. aureus*	0.26
10.0	500	88.25	87.65	88.25	88.85	85.10	86.35	85.85	*S. aureus*	0.23
		88.30	87.65	88.25	88.85	85.10	86.40	85.85	*S. aureus*	0.21
		88.50	87.75	88.60	89.00	85.40	86.60	86.30	*S. aureus*	0.26
5.00	250	88.35	87.75	88.35	88.90	85.25	86.40	85.90	*S. aureus*	0.27
		88.30	87.60	88.25	88.80	85.10	86.35	85.85	*S. aureus*	0.21
		88.60	87.85	88.70	89.05	85.60	86.70	86.25	*S. aureus*	0.24
2.50	125	88.40	87.65	88.30	88.90	85.25	86.35	85.90	*S. aureus*	0.29
		88.30	87.65	88.25	88.80	85.10	86.30	85.85	*S. aureus*	0.27
		88.50	87.65	88.60	88.90	85.55	86.60	86.15	*S. aureus*	0.31
1.25*1	62.5*1	88.40	87.65	88.40	88.90	85.20	86.30	85.90	*S. aureus*	0.35
		88.30	87.60	88.25	88.80	85.10	86.20	85.85	*S. aureus*	0.34
		88.60	87.75	88.65	89.00	85.50	86.50	86.25	*S. aureus*	0.37
0.625*2	31.3*2	88.40	N.D.	88.45	N.D.	N.D.	N.D.	86.10	Not identified	–
		88.30	N.D.	88.35	N.D.	85.20	N.D.	85.85	Not identified	–
		88.50	N.D.	88.65	N.D.	N.D.	N.D.	86.25	Not identified	–
0.313	15.6	88.40	N.D.	N.D.	N.D.	N.D.	N.D.	N.D.	Not identified	–
		N.D.	N.D.	N.D.	N.D.	N.D.	N.D.	86.00	Not identified	–
		N.D.	N.D.	N.D.	N.D.	N.D.	N.D.	N.D.	Not detected	–

***1**: limit of identification, ***2**: limit of detection (LOD)

c) *Klebsiella pneumoniae* (three independent experiments were performed in triplicate)

CFU/ PCR Tube	CFU/mL	Tm values							Tm mapping identification results	Difference Value
		Reg. 1	Reg. 2	Reg. 3	Reg. 4	Reg. 5	Reg. 6	Reg. 7		
10.0	500	90.05	89.35	89.95	89.60	86.45	87.45	86.45	*K. pneumoniae*	0.26
		90.25	89.75	90.15	89.85	86.60	87.50	86.60	*K. pneumoniae*	0.27
		90.20	89.60	90.20	89.85	86.70	87.70	86.65	*K. pneumoniae*	0.24
5.00	250	89.95	89.35	89.85	89.60	86.45	87.35	86.35	*K. pneumoniae*	0.20
		90.20	89.70	90.10	90.05	86.60	87.45	86.55	*K. pneumoniae*	0.23
		90.25	89.65	90.15	89.95	86.75	87.65	86.65	*K. pneumoniae*	0.17
2.50	125	89.90	89.50	89.90	89.50	86.40	87.25	86.25	*K. pneumoniae*	0.30
		90.25	89.80	90.15	89.85	86.50	87.50	86.50	*K. pneumoniae*	0.32
		90.25	89.75	90.20	89.85	86.70	87.60	86.60	*K. pneumoniae*	0.23
1.25	62.5	90.05	89.65	90.00	89.55	86.50	87.40	86.40	*K. pneumoniae*	0.37
		90.15	89.80	90.15	89.85	86.60	87.50	86.60	*K. pneumoniae*	0.32
		90.25	89.80	90.15	89.80	86.65	87.60	86.50	*K. pneumoniae*	0.29
0.625*1	31.3*1	90.00	89.65	89.90	89.55	86.40	87.30	86.30	*K. pneumoniae*	0.37
		90.25	89.90	90.15	89.85	86.65	87.50	86.60	*K. pneumoniae*	0.37
		90.35	90.00	90.25	89.90	86.65	87.60	86.65	*K. pneumoniae*	0.41
0.313*2	15.6*2	90.25	N.D.	90.25	N.D	86.65	N.D.	N.D	Not identified	–
		N.D.	N.D.	90.15	N.D.	86.60	N.D.	86.65	Not identified	–
		90.35	N.D.	90.25	N.D.	N.D.	N.D.	N.D.	Not identified	–
0.157	7.8	N.D.	N.D.	90.15	N.D.	N.D.	N.D.	N.D.	Not identified	–
		N.D.	N.D.	N.D.	N.D.	N.D.	N.D.	N.D.	Not detected	–
		N.D.	N.D.	N.D.	N.D.	N.D.	N.D.	N.D.	Not detected	–

***1**: limit of identification, ***2**: limit of detection (LOD)

Supplemental Table S7

List of bacterial colonies used in Table 2

140 colonies (51 bacterial species)

Bacterial colonies	No. of trials	Bacterial colonies	No. of trials
Escherichia coli	25	*Acinetobacter calcoaceticus*	1
Staphylococcus epidermidis	13	*Achromobacter xylosoxidans*	1
Klebsiella pneumoniae	8	*Aerococcus christensenii*	1
Staphylococcus aureus	5	*Bacteroides salyersiae*	1
Staphylococcus haemolyticus	5	*Bacteroides thetaiotaomicron*	1
Bacillus cereus	4	*Corynebacterium macginleyi*	1
Citrobacter freundii	4	*Enterococcus avium*	1
Enterobacter cloacae	4	*Enterococcus casseliglavus*	1
Finegoldia magna	4	*Eubacterium limosum*	1
Staphylococcus capitis	4	*Halomonas venusta*	1
Streptococcus agalactiae	4	*Prevotella bivia*	1
Enterococcus faecalis	3	*Prevotella intermedia*	1
Prevotella melaninogenica	3	*Prevotella nigrescens*	1
Pseudomonas aeruginosa	3	*Prevotella timonensis*	1
Staphylococcus hominis	3	*Prevotella veroralis*	1
Stenotrophomonas maltophilia	3	*Proteus mirabilis*	1
Anaerococcus vaginalis	2	*Serratia marcescens*	1
Bacteroides fragilis	2	*Staphylococcus cohnii*	1
Campylobacter jejuni	2	*Staphylococcus lugdunensis*	1
Clostridium perfringens	2	*Staphylococcus pettenkoferi*	1
Enterococcus faecium	2	*Streptococcus anginosus*	1
Klebsiella oxytoca	2	*Streptococcus constellatus*	1
Morganella morganii	2	*Streptococcus dysgalactiae*	1
Pasteurella multocida	2	*Streptococcus infantarius*	1
Pseudomonas putida	2	*Streptococcus mitis*	1
Staphylococcus caprae	2		

Supplemental Table S8

List of Tm mapping results of broad matches with the sequencing results in Table 2

Sequencing results	Identification results using the Tm mapping method					
	Diff. 1	Most similar bacteria	Diff. 2	2nd most similar bacteria	Diff. 3	3rd most similar bacteria
Staphylococcus haemolyticus	0.19	*Staphylococcus aureus*	0.24	*Staphylococcus haemolyticus*	0.51	*Staphylococcus lugdunensis*
Staphylococcus aureus	0.28	*Staphylococcus aureus*	0.28	*Staphylococcus haemolyticus*	0.48	*Staphylococcus lugdunensis*
Staphylococcus haemolyticus	0.37	*Staphylococcus haemolyticus*	0.37	*Staphylococcus warneri*	0.38	*Staphylococcus aureus*

Diff. = Difference Value

Supplemental Table S9

Blind test results for bacteria with similar Tm mapping shapes (Difference Value ≤ 0.28 shown by asterisks in Table S4) in the database

Bacterial DNA	Triplicate	Identification results using the Tm mapping method					
		Diff. 1	Most similar bacteria	Diff. 2	2nd most similar bacteria	Diff. 3	3rd most similar bacteria
Bacteroides dorei	**1**	0.20	*Bacteroides dorei*	0.37	*Bacteroides vulgatus*	0.85	*Bacteroides fragilis*
	**2**	0.23	*Bacteroides dorei*	0.42	*Bacteroides vulgatus*	1.10	*Bacteroides fragilis*
	**3**	0.26	*Bacteroides dorei*	0.47	*Bacteroides vulgatus*	1.08	*Bacteroides fragilis*
Bacteroides vulgatus	**1**	0.09	*Bacteroides vulgatus*	0.30	*Bacteroides dorei*	0.91	*Bacteroides fragilis*
	**2**	0.15	*Bacteroides vulgatus*	0.36	*Bacteroides dorei*	0.91	*Bacteroides fragilis*
	**3**	0.20	*Bacteroides vulgatus*	0.48	*Bacteroides dorei*	0.96	*Bacteroides fragilis*

Diff. = Difference Value

Supplemental Table S10

Comparison of the cell count using flow cytometry method and the colony count using conventional culture method starting from the same bacterial suspensions

E. coli* suspensions	**Cell count using flow cytometry** (cells/µL)	**Colony count of culture dishes** (CFU/µL)	**Percentage of colony formation** (CFU/cells)
E. coli suspension # 1	1081	898	83.1 %
E. coli suspension # 2	971	853	87.8 %
E. coli suspension # 3	1147	1018	88.8 %

*****: Before performing the tests, *Escherichia coli* (ATCC25922) was cultivated in Muller-Hinton Broth at 37°C for 12 hours, then bacterial suspensions were prepared at a concentration of around 1000 bacteria/µL. Each suspension was prepared separately.

Supplemental Table S11

Quantification of approximate bacterial concentrations in the blood of patients with sepsis

DNA templates (2 µL/20 µL of PCR reaction mixture)		Ct*2 averages from triplicate experiments	16S ribosomal RNA operon copy number	Blood bacterial concentrations (CFU/mL)
Control DNA (*E. coli*)	80 CFU/µL*1	9.77	7	8,000
	10 CFU/µL*1	13.84	7	1,000
Patient # 1 (*E. coli*)		15.66	7	395
Patient # 2 (*E. coli*)		5.44	7	73,200
Patient # 3 (*S. aureus*)		8.93	5	17,220
Patient # 4 (*S. aureus*)		5.10	5	121,800
Patient # 5 (*S. epidermidis*)		10.05	5	9,716
Patient # 6 (*K. pneumoniae*)		10.84	8	4,051

***1**: Concentration in a DNA elution buffer. Bacterial DNA was eluted with 100 µL of elution buffer, and 2µL of which was used as a DNA template for one PCR reaction.

***2**: The Ct (threshold cycle) values amplified by Region 3 primers in the second nested PCR were analyzed using the Rotor-Gene Q software program.

We did quantitative PCR analysis for relative quantification of bacteria in the blood of patients with sepsis. The pathogenic bacteria had already been identified by both the Tm mapping method and the conventional culture method. In this experiment, the amplification efficiency was 66.7% per PCR cycle, which depends on the enzyme activity of the thermostable DNA polymerase, and the blood bacterial concentrations were finally adjusted by the 16S ribosomal RNA operon copy number.

Supplemental Figure S1

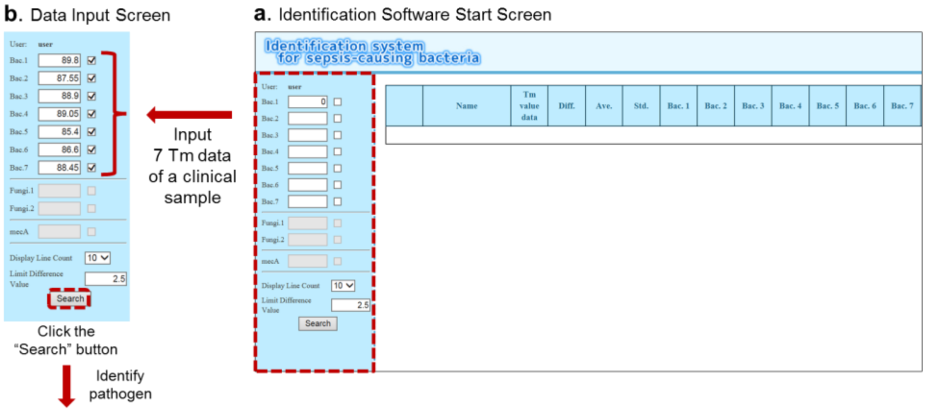

c. Identification Results

Diff. = Difference Values

	Name	Tm value data	Diff.	Ave.	Std.	Bac. 1	Bac. 2	Bac. 3	Bac. 4	Bac. 5	Bac. 6	Bac. 7
d Info	Enterococcus faecalis	1	0.12	88.95		90.75	88.60	89.93	90.00	86.43	87.58	89.38
Info	Enterococcus faecalis	2	0.35	88.49		90.10	88.10	89.50	89.60	86.15	87.10	88.85
Info	Enterococcus faecium	1	0.96	88.54		90.15	87.85	89.75	89.50	86.75	87.00	88.75
Info	Enterococcus faecium	2	0.98	88.49		90.00	87.75	89.50	89.50	86.75	87.15	88.75
Info	Granulicatella adiacens	2	1.01	88.64		91.10	88.25	89.50	89.25	85.90	87.75	88.75
Info	Granulicatella adiacens	1	1.05	88.64		91.10	88.35	89.50	89.35	85.85	87.75	88.60
Info	Lactobacillus crispatus	2	1.14	88.29		89.35	87.75	89.10	89.40	86.10	86.85	89.50
Info	Lactobacillus crispatus	1	1.24	88.29		89.15	87.75	89.15	89.40	86.15	87.00	89.40
e Info	Enterococcus avium	1	1.27	87.79		89.15	87.15	88.85	88.85	86.25	86.50	87.75
Info	Enterococcus avium	2	1.28	87.81		89.10	87.25	88.85	89.00	86.25	86.50	87.75

d. Shape Comparison and Information about the Identified Pathogen

Bacteria name

Enterococcus faecalis

Microbiology

Gram-positive, aerobic cocci. A part of normal human gastrointestinal flora. Also can be found in soil, food, and water.

Clinical Syndromes

Has relatively low virulence, but very resistant to antimicrobial therapy. Can cause urinary tract infection, soft tissue infection, intraabdominal infection, endocarditis, and line associated blood stream infection. Colonization of resistant strain is a major problem in infection control.

Selecting a therapeutic agent

Wild types are susceptible to ampicillin, vancomycin, linezolid, and daptomycin. Aminoglycosides can have synergistic effect with cell-wall acting agents, but high-level resistance has to be ruled out. Cephalosporin does not have any activity. Resistance to ampicillin and vancomycin (i.e. VRE) has been a huge problem, especially in nosocomial settings.

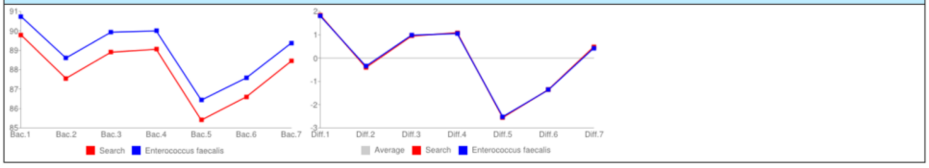

Comparison of raw data Adjusted by averages

e. Shape Comparison and Information about other Pathogens

Bacteria name

Enterococcus avium

Microbiology

Gram-positive, aerobic cocci. Most commonly associated with birds, but can be found in soil, food, and water.

Clinical Syndromes

Rarely a human pathogen. Has relatively low virulence, but very resistant to antimicrobial therapy. Can cause urinary tract infection, soft tissue infection, intraabdominal infection, endocarditis, and line associated blood stream infection.

Selecting a therapeutic agent

Wild types are susceptible to ampicillin, vancomycin, linezolid, and daptomycin. Aminoglycosides can have synergistic effect with cell-wall acting agents, but high-level resistance has to be ruled out. Cephalosporin does not have any activity. Resistance to ampicillin and vancomycin (i.e. VRE) has been a huge problem, especially in nosocomial settings.

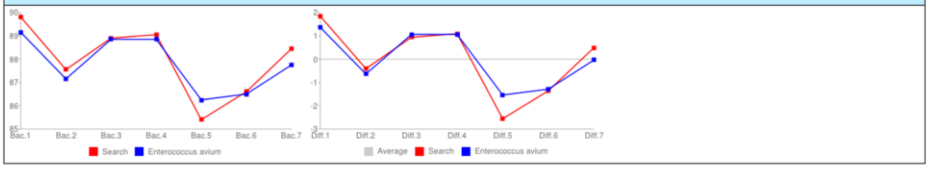

Identification software program on the web

Using the identification software program available on the web, any user can identify bacterial isolates without additional training.

(**a**) Any user can access the identification software program via the internet.

(**b**) First, the user inputs the seven Tm values for a clinical sample and then clicks the “search” button.

(**c**) The identification results are shown promptly. The results are lined up in ascending order according to the Difference Values.

(**d**) Next, the user clicks the “Info” button, where information about the pathogen is provided. It is also possible to compare the raw data to the Tm mapping shape in the database.

(**e**) Information about other pathogens can be seen as well, in addition to the differences between the shapes.

Supplemental Figure S2

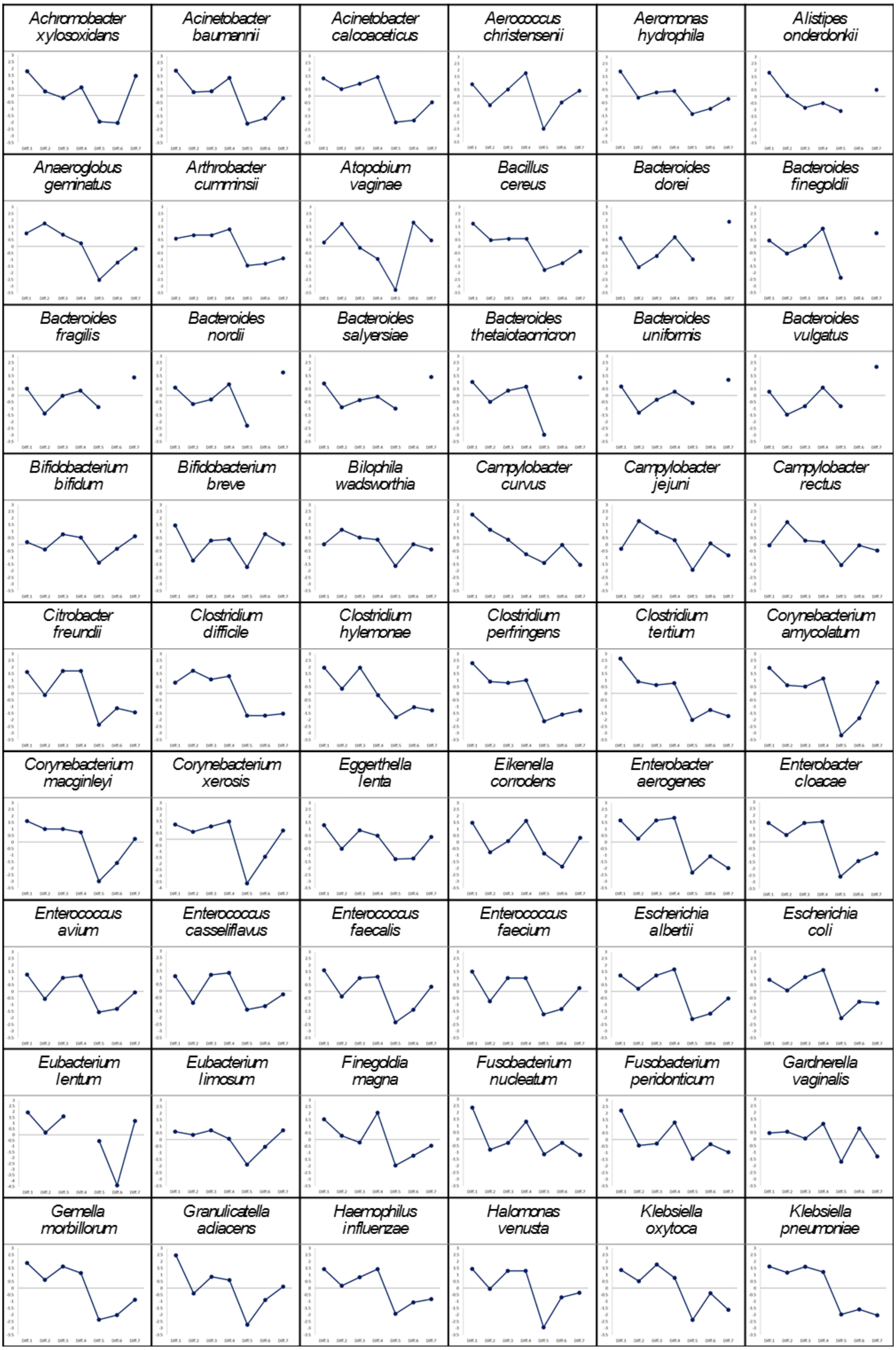

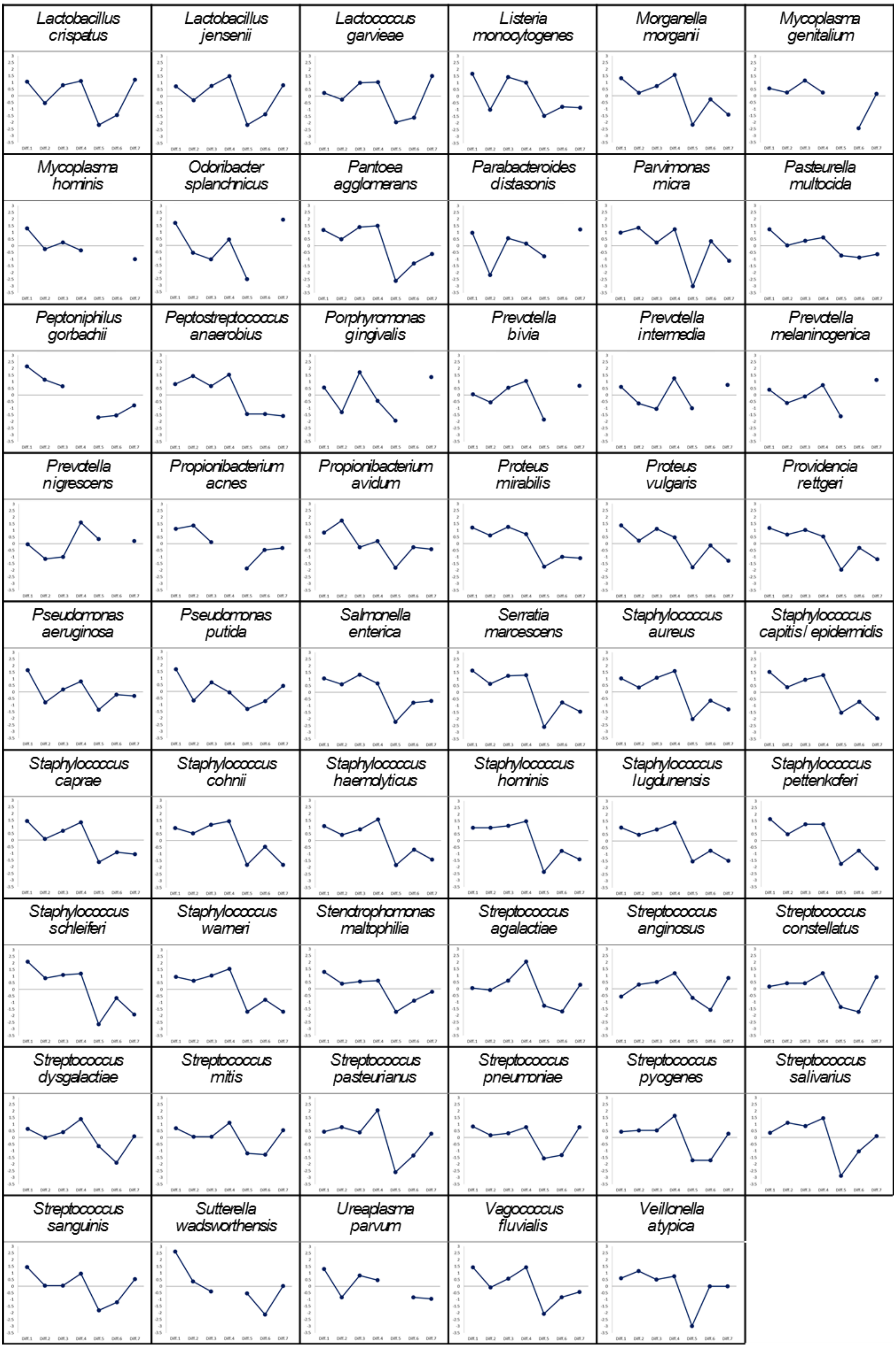
Lactobacillus crispatus
Lactobacillus jensenii
Lactococcus garvieae
Listeria monocytogenes
Morganella morganii
Mycoplasma genitalium
Mycoplasma hominis
Odoribacter splanchnicus
Pantoea agglomerans
Parabacteroides distasonis
Parvimonas micra
Pasteurella multocida
Peptoniphilus gorbachii
Peptostreptococcus anaerobius
Porphyromonas gingivalis
Prevotella bivia
Prevotella intermedia
Prevotella melaninogenica
Prevotella nigrescens
Propionibacterium acnes
Propionibacterium avidum
Proteus mirabilis
Proteus vulgaris
Providencia rettgeri
Pseudomonas aeruginosa
Pseudomonas putida
Salmonella enterica
Serratia marcescens
Staphylococcus aureus
Staphylococcus capitis / epidermidis
Staphylococcus caprae
Staphylococcus cohnii
Staphylococcus haemolyticus
Staphylococcus hominis
Staphylococcus lugdunensis
Staphylococcus pettenkoferi
Staphylococcus schleiferi
Staphylococcus warneri
Stenotrophomonas maltophilia
Streptococcus agalactiae
Streptococcus anginosus
Streptococcus constellatus
Streptococcus dysgalactiae
Streptococcus mitis
Streptococcus pasteurianus
Streptococcus pneumoniae
Streptococcus pyogenes
Streptococcus salivarius
Streptococcus sanguinis
Sutterella wadsworthensis
Ureaplasma parvum
Vagococcus fluvialis
Veillonella atypica

The Tm mapping shapes of the 107 species of bacteria registered in the database

The X-axis shows the average of the seven Tm values.

Supplemental Figure S3

a

Digital Filter: **ON**

**Reg. 1**	Tm values		Melting Peak Curve
	single	mixed	
E. coli	89.25	88.75	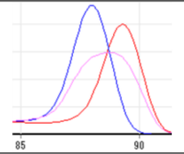
S. aureus	88.00	88.75	

Digital Filter: **OFF**

**Reg. 1**	Tm values		Melting Peak Curve
	single	mixed	
E. coli	89.40	89.40	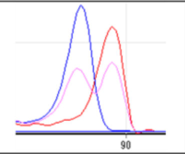
S. aureus	88.10	88.00	

Digital Filter: **ON**

**Reg. 2**	Tm values		Melting Peak Curve
	single	mixed	
E. coli	88.50	88.15	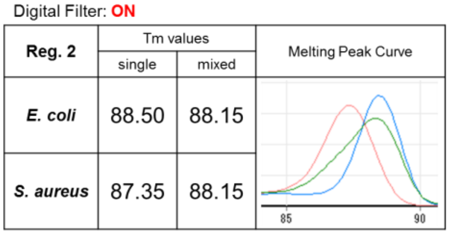
S. aureus	87.35	88.15	

Digital Filter: **OFF**

**Reg. 2**	Tm values		Melting Peak Curve
	single	mixed	
E. coli	88.60	88.65	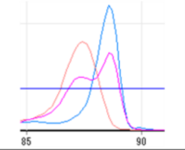
S. aureus	87.50	87.35	

Digital Filter: **ON**

**Reg. 3**	Tm values		Melting Peak Curve
	single	mixed	
E. coli	89.40	88.75	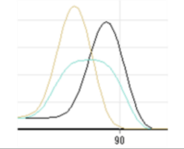
S. aureus	88.10	88.75	

Digital Filter: **OFF**

**Reg. 3**	Tm values		Melting Peak Curve
	single	mixed	
E. coli	89.50	89.50	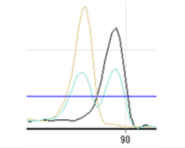
S. aureus	88.15	88.10	

Digital Filter: **ON**

**Reg. 4**	Tm values		Melting Peak Curve
	single	mixed	
E. coli	89.90	89.00	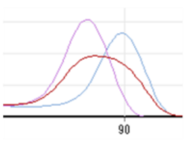
S. aureus	88.65	89.00	

Digital Filter: **OFF**

**Reg. 4**	Tm values		Melting Peak Curve
	single	mixed	
E. coli	90.10	90.10	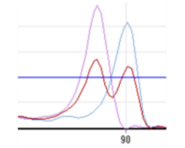
S. aureus	88.75	88.75	

Digital Filter: **ON**

**Reg. 5**	Tm values		Melting Peak Curve
	single	mixed	
E. coli	86.35	86.15	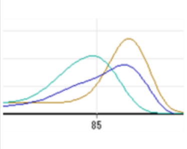
S. aureus	84.85	86.15	

Digital Filter: **OFF**

**Reg. 5**	Tm values		Melting Peak Curve
	single	mixed	
E. coli	86.50	86.60	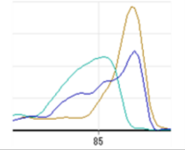
S. aureus	85.25	84.25	

Digital Filter: **ON**

**Reg. 6**	Tm values		Melting Peak Curve
	single	mixed	
E. coli	87.75	86.40	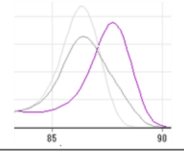
S. aureus	86.35	86.40	

Digital Filter: **OFF**

**Reg. 6**	Tm values		Melting Peak Curve
	single	mixed	
E. coli	87.85	87.85	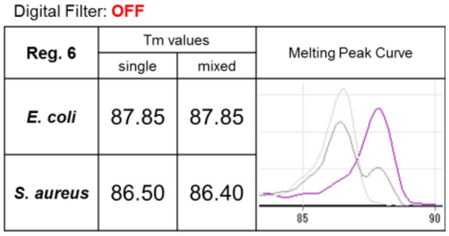
S. aureus	86.50	86.40	

Digital Filter: **ON**

**Reg. 7**	Tm values		Melting Peak Curve
	single	mixed	
E. coli	87.50	87.40	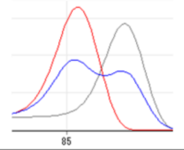
S. aureus	85.50	85.35	

Digital Filter: **OFF**

**Reg. 7**	Tm values		Melting Peak Curve
	single	mixed	
E. coli	87.60	87.40	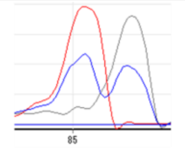
S. aureus	86.00	86.00	

b

Digital Filter: ON — Diff. = Difference Values

Bacterial DNA	sample	Tm mapping identification	Diff.
E. coli	single	*Escherichia coli*	0.19
	mixed	*Escherichia albertii*	0.55
S. aureus	single	*Staphylococcus aureus*	0.21
	mixed	*S. capitis / epidermidis*	0.67

Digital Filter: OFF — Diff. = Difference Values

Bacterial DNA	sample	Tm mapping identification	Diff.
E. coli	single	*Escherichia coli*	0.15
	mixed	*Escherichia coli*	0.31
S. aureus	single	*Staphylococcus aureus*	0.22
	mixed	*Escherichia coli*	0.73

Tm mapping results for a mixed sample that contains equal amounts of two species of bacteria (*Escherichia coli* and *Staphylococcus aureus*)

Using a mixed sample containing equal amounts of *E. coli* and *S. aureus*, we performed Tm mapping identification. We used the RotorGeneQ instrument and measured the seven Tm values of the seven amplicons with a Digital Filter ON or OFF, which calculates the melting temperature by approximating the melting peak curve to the normal distribution curve (**a**). As a result, because the different Tm values overlapped, the equal amounts of the two species of bacteria interfered with each other's Tm mapping identification (**b**).

Reg. 1 – 7 = amplicons amplified by the Region 1 – 7 primers

Supplemental Figure S4

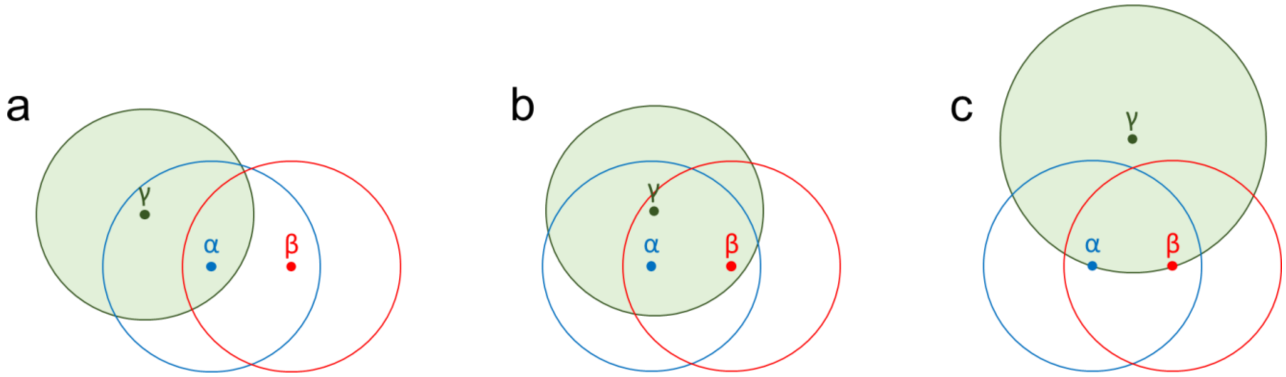

Concept of interference with the Tm mapping identification between bacteria with similar Tm mapping shapes

These figures (**a**, **b**, **c**) show seven-dimensional space. From a mathematic point of view, the Difference Value is the distance between two points in seven dimensions. In this setting, sample γ is the bacterial isolate from the patient sample that we wish to identify, and point γ is the Tm mapping result. Bacteria α is the pathogenic bacteria, and bacteria β is the bacteria with a similar Tm mapping shape (both Difference Values are less than 0.28). Point α is bacteria α's Tm mapping data in the database, and point β is bacteria β's Tm mapping data in the database. The blue and red circles show the Difference Value boundaries (0.28).

(**a**) The green circle shows the Difference Value boundary (0.28) of sample γ. The Tm mapping result is bacteria α (Difference Value = 0.26). In this case, bacteria β does not interfere with the Tm mapping identification. The distance between the two points in seven dimensions provides enough

diversity so that bacteria β does not interfere with the Tm mapping identification.

(**b**) The green circle shows the Difference Value boundary (0.28) of sample γ. The Tm mapping result is bacteria α (Difference Value = 0.20) or bacteria β (Difference Value = 0.27). In this case, bacteria β interferes with the Tm mapping identification, such that the result cannot be narrowed down to one bacterial species.

(**c**) In this case, because sample γ is a mutant strain or one dominant strain in a polymicrobial infection, the green circle shows a Difference Value boundary of 0.37. The Tm mapping result is bacteria α (Difference Value = 0.37) or bacteria β (Difference Value = 0.37). In this case (0.28 < Difference Value ≤ 0.5), the identification result with the lowest Difference Value is highly likely to be the bacterial isolate (**Table 1**), which means either bacteria α or bacteria β.

Supplemental Figure S5

a

Amplicon	*E. faecalis* DNA diluted with distilled water	Melting temperature
Amplicon amplified by Region 3 primers	undiluted	89.75
	10-fold	89.85
	100-fold	89.90
	1000-fold	89.85
	10000-fold	89.90

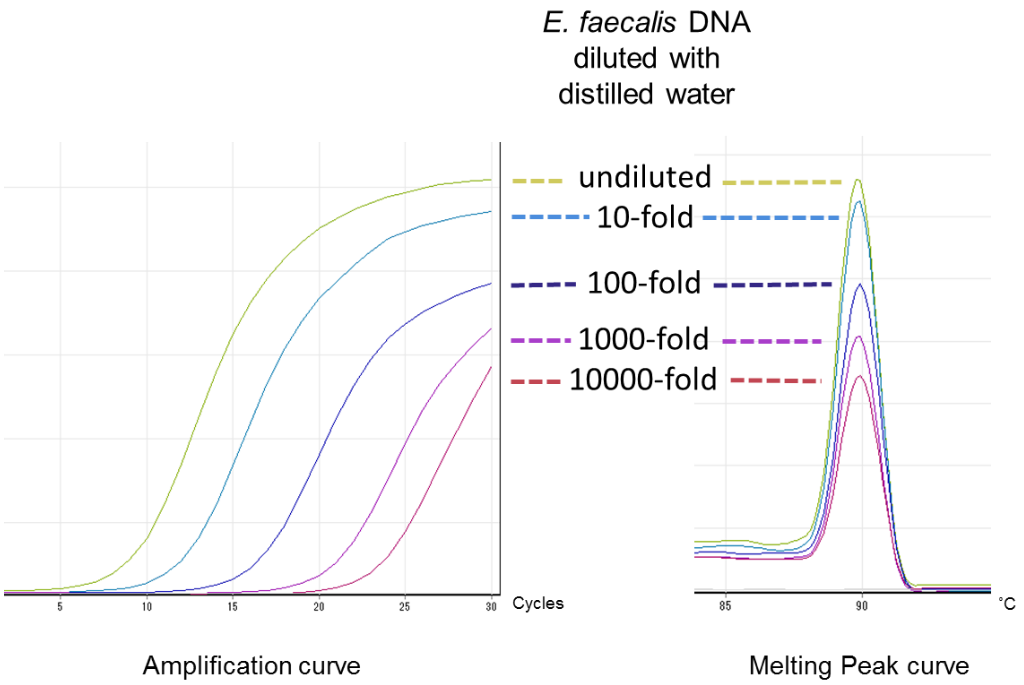

b

E. coli DNA diluted with distilled water	Melting Temperature (Duplicate)						
	Reg. 1	Reg. 2	Reg. 3	Reg. 4	Reg. 5	Reg. 6	Reg. 7
undiluted	89.35	88.60	89.50	90.00	86.50	87.75	87.60
	89.40	88.60	89.40	90.00	86.40	87.75	87.60
100-fold	89.35	88.65	89.50	90.00	86.40	87.75	87.60
	89.35	88.60	89.40	90.00	86.40	87.75	87.50
10000-fold	89.35	88.65	89.50	90.00	86.35	87.75	87.60
	89.35	88.60	89.50	89.90	86.40	87.75	87.50

Reg. 1 – 7 = Region 1 – 7 primers

Validation of the melting temperatures of the same amplicon using different DNA concentrations

(**a**) The *Enterococcus faecalis* DNA template was diluted from 10 to 10,000-fold with molecular-grade distilled water and amplified using the Region 3 primers. Finally, the melting temperatures of the undiluted and diluted amplicons (five different DNA concentrations) were measured in the same trial.

(**b**) The *E. coli* DNA template was diluted from 100 to 10,000-fold with molecular-grade distilled water and then amplified using the Region 1 - 7 primers, respectively. Finally, the melting temperatures of the undiluted and diluted amplicons (three different DNA concentrations) were measured in the same trial.
